# Sleep neuroimaging: Review and future directions

**DOI:** 10.1111/jsr.14462

**Published:** 2025-02-12

**Authors:** Mariana Pereira, Xinyuan Chen, Anastasiya Paltarzhytskaya, Yibran Pacheсo, Nils Muller, Leonore Bovy, Xu Lei, Wei Chen, Haoran Ren, Chen Song, Laura D. Lewis, Thien Thanh Dang‐Vu, Michael Czisch, Dante Picchioni, Jeff Duyn, Philippe Peigneux, Enzo Tagliazucchi, Martin Dresler

**Affiliations:** ^1^ Donders Institute of Cognition and Behaviour Radboud University Medical Center Nijmegen Netherlands; ^2^ Sleep and NeuroImaging Center, Faculty of Psychology Southwest University Chongqing China; ^3^ School of Information Science and Technology & Human Phenome Institute Fudan University Shanghai China; ^4^ School of Health and Engineering University of Shanghai for Science and Technology Shanghai China; ^5^ Cardiff University Brain Research Imaging Centre, School of Psychology Cardiff University Cardiff UK; ^6^ Department of Biomedical Engineering Boston University Boston Massachusetts USA; ^7^ Center for Systems Neuroscience Boston University Boston Massachusetts USA; ^8^ Department of Health, Kinesiology and Applied Physiology Concordia University & Centre de recherche de l'Institut universitaire de gériatrie de Montréal (CRIUGM) Montreal Quebec Canada; ^9^ Max Planck Institute of Psychiatry Munich Germany; ^10^ Advanced Magnetic Resonance Imaging Section National Institute of Neurological Disorders and Stroke Bethesda Maryland USA; ^11^ Advanced MRI Section, Laboratory of Functional and Molecular Imaging, National Institute of Neurological Disorders and Stroke National Institutes of Health Bethesda Maryland USA; ^12^ UR2NF – Neuropsychology and Functional Neuroimaging Research Unit at CRCN – Centre de Recherches Cognition et Neurosciences, and UNI – ULB Neuroscience Institute Université libre de Bruxelles (ULB) Brussels Belgium; ^13^ Departamento de Física Universidad de Buenos Aires and Instituto de Física de Buenos Aires Buenos Aires Argentina; ^14^ Latin American Brain Health Institute Universidad Adolfo Ibanez Santiago Chile

**Keywords:** functional connectivity, functional magnetic resonance imaging, neuroimaging, positron emission tomography, regional cerebral blood flow, sleep

## Abstract

Sleep research has evolved considerably since the first sleep electroencephalography recordings in the 1930s and the discovery of well‐distinguishable sleep stages in the 1950s. While electrophysiological recordings have been used to describe the sleeping brain in much detail, since the 1990s neuroimaging techniques have been applied to uncover the brain organization and functional connectivity of human sleep with greater spatial resolution. The combination of electroencephalography with different neuroimaging modalities such as positron emission tomography, structural magnetic resonance imaging and functional magnetic resonance imaging imposes several challenges for sleep studies, for instance, the need to combine polysomnographic recordings to assess sleep stages accurately, difficulties maintaining and consolidating sleep in an unfamiliar and restricted environment, scanner‐induced distortions with physiological artefacts that may contaminate polysomnography recordings, and the necessity to account for all physiological changes throughout the sleep cycles to ensure better data interpretability. Here, we review the field of sleep neuroimaging in healthy non‐sleep‐deprived populations, from early findings to more recent developments. Additionally, we discuss the challenges of applying concurrent electroencephalography and imaging techniques to sleep, which consequently have impacted the sample size and generalizability of studies, and possible future directions for the field.

## 1 INTRODUCTION

From the first to our last day, sleep is a constant that marks the passing of time. There is probably no single activity we spend more time doing, but despite that it still remains a puzzle throughout the history of humankind. The invention of electroencephalography (EEG) made it possible to monitor the brain's electrical activity non‐invasively, leading to ground‐breaking findings in early sleep research such as the discovery of rapid eye movements (REM) and cyclic nocturnal sleep behaviour. Later discoveries identified neurophysiological events within sleep stages such as sleep spindles and slow waves as a marker of non‐REM (NREM) sleep, or ponto‐geniculo‐occipital (PGO) waves, traceable in animal models, during REM sleep (for review, see Dresler et al., 2014). The human sleep cycle consists of regular progress through a series of global brain states characterized by specific neurophysiological changes. NREM sleep is traditionally divided into three stages exhibiting distinct EEG patterns: light NREM sleep, or stage N1, marks the transition from wakefulness to sleep, with low‐amplitude theta waves (4–7 Hz), slow rolling eye movements and lower muscle activity than wakefulness; stage N2 features hallmark sleep spindles (brief bursts of 12–14 Hz activity) and K‐complexes (large, isolated slow waves); and stage N3, or slow‐wave sleep (SWS), is characterized by high‐amplitude, low‐frequency delta waves (0.5–3 Hz), often associated with restorative processes. REM sleep, in contrast, is defined by low‐amplitude mixed‐frequency EEG activity featuring rapid eye movements and even lower muscle activity. Physiologically, eye movements, muscle tone and autonomic activity further differentiate sleep stages, with muscle tone progressively decreasing through NREM stages and reaching near‐complete atonia during REM sleep, alongside irregular heart rate and respiration. A typical sleep episode starts with light NREM sleep, progressing to deeper stages, and finally to REM sleep. Still, individuals do not remain in REM sleep but rather cycle between stages of NREM and REM throughout the night. NREM sleep constitutes about 75% of total time spent in sleep, whereas REM sleep constitutes the remaining 25%. In healthy adults, NREM sleep accounts for the majority of sleep time in the first half of the night, as REM sleep increases as the night progresses and accounts for much of the sleep time in the second half of the night (Carskadon & Dement, 2005). Although animal research has advanced sleep characterization across species, several pivotal questions on why and how human beings sleep are still to be addressed. Brain imaging has played a role in understanding brain function and metabolism during sleep and associated sleep‐specific features. Advances in neuroimaging may yield questions that remained unsolved and need addressing, to name a few examples, the relationship between sleep and brain clearance, the causal relation with neurodegenerative diseases, and the possible functions of dreaming.

Neuroimaging can be defined as any technique capable of imaging the anatomy, function or physiology of the brain. In this review, we will focus on positron emission tomography (PET), and structural and functional magnetic resonance imaging (sMRI and fMRI), but also briefly mention other neuroimaging techniques. The concept of tomographic imaging (Phelps et al., 1975; Ter‐Pogossian et al., 1975) and the development of positron‐emitting radiopharmaceuticals (Ido et al., 1978) led to the development of the PET technology. PET imaging makes use of radioactive tracers to measure and visualize physiological information, such as glucose consumption, dopamine release and blood flow (tissue perfusion) in the body. By detecting radioactive decay as an accumulated component in specific brain regions, H_2_^15^O PET imaging reveals the amount of blood flow across the whole brain, thus providing an indirect marker of neuronal activity. The increased ^15^oxygen‐labelled (H_2_^15^O) concentration in a brain area reflects locally increased (regional) cerebral blood flow (rCBF) resulting from higher energetic demands due to increased neural activity. Similarly, locally increased glucose‐labelled (fluorodeoxyglucose or FDG‐18) consumption reflects the energetic neuronal needs in the corresponding area, glucose and oxygen being the two primary sources of energy for brain activity (note that only a single image is acquired reflecting the time‐averaged energy consumption).

Structural MRI takes advantage of the abundance of hydrogen atoms in biological organisms, particularly in water and fat. This method aligns the spins of hydrogen atoms using a large magnetic field, typically 1.5 or 3 T in human studies. After the spins orientations are perturbed using a radiofrequency pulse at the nuclear magnetic resonance (NMR) Larmor frequency, they precess around their axis (which generates the measurable signal), and dephase and realign with the magnetic field at different time rates determined by the local tissue properties. The decay rates are exploited to distinguish different brain tissues in sMRI. Contrast in diffusion‐weighted MRI (DWI) relies on the microscopic movement of water molecules, as the brain's various tissue types and geometries will affect the diffusive motion of water molecules in specific ways. A particular class of diffusion MRI is diffusion tensor imaging (DTI), which promises to characterize microstructural changes (Campbell & Pike, 2019). For instance, DTI is used to characterize the degree of anisotropy (fractional anisotropy), the orientation of directional diffusion (radial and axial diffusivity D_r_/D_a_), or magnitude characterization (mean diffusivity; for more in‐depth details on DTI measures estimation, please see Alexander et al., 2007; Assaf & Basser, 2005; Stee & Peigneux, 2021; Zhang et al., 2012 for reviews).

Functional MRI has become the most widely used technique for studying human cognitive processes since its development in the early 1990s (Kwong et al., 1992). Blood oxygenation level‐dependent (BOLD) fMRI measures changes in the total amount of deoxygenated haemoglobin in a voxel over time, and the quantity of deoxygenated haemoglobin depends not just on the extraction of oxygen by active neurons but also on blood flow and blood volume changes that together shape the BOLD haemodynamic response (Huettel et al., 2004). The BOLD signal primarily reflects the input and intracortical processing in a given region, rather than the output reflected in action potential firing. Compared with PET imaging, fMRI is non‐invasive and can be repeatedly used in a broader range of individuals without the safety constraints of PET regarding radiation exposure. Finally, fMRI allows better spatial and temporal resolution (down to ~1 mm and a few seconds, respectively; Table 1), allowing the imaging of brain activity changes taking place over much shorter time spans closer to the dynamics of cognitive processes. Combined, these advantages explain fMRI's rapidly expanding application in cognitive neuroscience.

**TABLE 1:** Comparison between neuroimaging methods for human sleep research.

Modality	Principle	Space resolution	Acquisition time	Information	Cost	Environment and requirements
SPECT	Radiation level from gamma‐emitting injected blood‐injected tracers	6–8 mm	Minutes	rCBF (perfusion)	Medium	Injection of radioactive tracer
PET	Gamma radiation level secondary to positron emission from blood‐injected tracers	2–3 mm	Seconds–Minutes	rCBF (perfusion)	High	Injection of radioactive tracer
fNIRS	Blood oxygenation and blood volume dependent absorption of near‐infrared light	Centimetre	Seconds	CBV, blood oxygenation	Low	Required light avoidance
fMRI	Blood deoxyhaemoglobin concentration‐dependent NMR relaxation	1–3 mm^a^	Seconds^a^	Blood flow (vascular)	High	Required fixed head, in‐scanner space limitation, acoustic noise. Disturbs EEG acquisition
sMRI	Density and NMR relaxation properties of water protons	0.5–1 mm	Seconds–Minutes	Tissue composition	High	Required fixed head, in‐scanner space limitation, acoustic noise. Disturbs EEG acquisition
DWI/DTI	Water diffusion based on tissue structural properties	1–3 mm	Minutes	Tissue structure	High	Required fixed head, in‐scanner space limitation, acoustic noise. Disturbs EEG acquisition

Abbreviations: CBV, cerebral blood volume; DWI/DTI, diffusion‐weighted imaging/diffusion tensor imaging; EEG, electroencephalography; fMRI, functional magnetic resonance imaging; fNIRS, functional near‐infrared spectroscopy; NMR, nuclear magnetic resonance; PET, positron emission tomography; rCBF, regional cerebral blood flow; sMRI, structural magnetic resonance imaging; SPECT, single‐photon emission computed tomography.

^a^ These values are based on typical sleep neuroimaging studies; however, modern fMRI can achieve higher spatiotemporal resolution.

For sleep neuroimaging studies, the various features of each image acquisition technique not only determine the quality of the image itself but the success of the study, since it is necessary to have an adequate environment for the participants to consolidate and maintain sleep including its deeper stages (Table 1). For instance, the different tracers used in PET imaging have different kinetics, resulting in H_2_^15^O shorter half‐life allowing repeated measurements at the same night, whereas the longer half‐life of FDG‐18 allows only a single acquisition per night. However, the latter has the advantage that once the injection takes place during the night, imaging acquisition can be performed during awakening, thus enabling the subjects to sleep more comfortably in a bed. The complementary strengths of each method make simultaneous EEG and neuroimaging recordings crucial for sleep neuroimaging studies. EEG accurately identifies sleep stages and their characteristics using standard polysomnographic classification, while neuroimaging provides insight into brain activity and metabolism with superior spatial resolution. This combined approach allows novel interpretations of event‐related activity or sleep stages time‐locked to brain dynamics. However, integrating the two methods, especially in the case of fMRI, requires careful attention, as all EEG equipment must be non‐magnetic to ensure safety and signal quality. A few example challenges for sleep research include the technical aspects impacting the final generalizability of results and data quality. For example, the MRI environment is extremely loud and uncomfortable for participants, often leading to inflated drop‐out rates and, consequently, smaller sample sizes compared with other neuroimaging studies. While the use of noise‐cancelling headphones and custom‐made earplugs are great mitigation strategies (discussed further in the Discussion section), the smaller sample sizes limit the generalizability of findings, and larger studies are needed to confirm initial results while better representing the broader population. Additionally, technical factors such as MRI gradients switching and the cardio‐ballistic artefacts affect EEG signal quality, further complicating data acquisition and interpretation (Table A1).

The literature selection for this review was based on a systematic search of the online databases PubMed, Web of Science and Google Scholar for English articles. The search terms included “sleep” and “neuroimaging” keywords present either in the title/abstract or abstract fields. The initial search returned a total of 1894 articles and, after removing duplicates and screening for non‐sleep‐deprived studies in healthy adults, the remaining articles were selected by researchers based on their relevance to the following research questions: “How has sleep neuroimaging been conducted?”, “What are the general neuroimaging discoveries to sleep research?”, and finally “What are the advances in the field?”. We decided to focus the review on two imaging modalities (PET and MRI) and two main aspects of sleep neuroimaging. First, we will summarize the results of sleep neuroimaging studies carried out in healthy non‐sleep‐deprived subjects. Secondly, we will examine the challenges associated with brain imaging in sleep research and discuss the potential future directions for the field. We will highlight the limitations and confounds that affect the interpretation of neuroimaging data, and explore emerging technologies and their potential applications in sleep research.

## 2 NEUROIMAGING OF SLEEP MACROSTRUCTURE

Neuroimaging techniques allow new insights into sleep macrostructure, allowing researchers to explore the neural activity and metabolic demands of the brain during different stages of sleep. This section covers two important subsections related to neuroimaging of sleep. The first one focuses on local changes in brain blood flow, which have been primarily measured using PET imaging. The results of these studies have shown that there are significant regional differences in brain activity and metabolic rate during the sleep–wake cycle, with decreases in activity observed during NREM sleep, and more heterogeneous activity observed during REM sleep. The second subsection discusses resting‐state networks and thalamocortical connectivity during sleep, which have been largely studied using fMRI. Researchers have observed changes in the integrity of the default mode network (DMN) during different stages of sleep, as well as alterations in thalamocortical functional connectivity. These findings provide valuable insights into the mechanisms underlying sleep stages.

### 2.1 Local changes in brain blood flow

Regional changes in blood flow by increases or accumulation of tracer components is a pivotal indirect means to measure brain neural activity and metabolic consumption. As a consequence, the assessment of changes in regional brain blood flow has advanced our understanding of neural activity and metabolic demands throughout the sleep–wake cycle. Early PET studies assessed cerebral glucose metabolic rates during sleep, measured by FDG‐18, in comparison to wakefulness. These studies showed a continuous reduction in metabolic rate from wakefulness to NREM sleep, being greater in frontal than temporal areas, and even more evident in the basal ganglia and thalamus compared with most of the cortex, whereas activity was at similar levels or even higher during REM sleep than in wakefulness, but more heterogeneous (Buchsbaum et al., 1989; Maquet et al., 1990). Activity further decreased from NREM light stage N2 to deep NREM sleep stage N3 (Maquet et al., 1992), suggesting a continuous process in the transition from wakefulness to deep NREM sleep. Using H_2_^15^O PET imaging, Maquet et al. (1996) more precisely delineated the structures in which rCBF is diminished during NREM sleep. Negative correlations within the mesencephalon and the dorsal pons during NREM or SWS were thought to reflect the decreasing neuronal firing of brainstem systems leading to the hyperpolarization of thalamic nuclei (Steriade & McCarley, 2013), eventually resulting in synchronized discharge patterns over large neuronal populations that generate the SWS hallmark's slow and high‐amplitude oscillations measured by the EEG. These findings suggest that rCBF distribution is not homogeneous during SWS. With the exception of primary cortical areas, secondary and associative cortical areas (more specifically in prefrontal and parietal regions) presented larger decreases than others, indicating that cellular processes occurring during SWS might be modulated differently in these regions. Likewise, Braun et al. (1997) and Andersson et al. (1998) observed a decrease in rCBF in the brainstem, thalamus and frontoparietal cortex, concluding that these areas play a role in the mediation of arousal. An increasingly widespread deactivation of cortical regions during the descent from light to deep NREM sleep was also observed (Kajimura et al., 1999). On the subcortical level, activity of the midbrain reticular formation was maintained during light but not deep NREM sleep, thus representing a key distinguishing correlate of sleep depth. Further, in agreement with previous PET studies (Maquet et al., 1990; Maquet et al., 1992), a significant decrease in rCBF as a function of delta activity was observed in the thalamus, the cerebellum and the frontal cortex, specifically at the anterior cingulate and orbitofrontal cortex (Hofle et al., 1997).

### 2.2 Resting‐state networks and thalamocortical connectivity

Functionally connected regions share information observed in correlated time series, forming connectivity patterns known as resting‐state functional networks. These networks have been broadly categorized into cognitive control, sensory systems (visual, auditory and sensorimotor) and the DMN, which characterizes brain activity in the absence of goal‐directed tasks, with much speculation about its integrity (stability) during sleep. The thalamocortical network plays a central role in sensory information processing, especially during states of arousal (Castro‐Alamancos, 2004). Thus, while thalamocortical connectivity is distinct from the arousal network, it is significantly influenced by it. Understanding the interplay between these networks is crucial for elucidating the mechanisms underlying brain function and dynamic changes within networks during sleep.

Despite the physiological and behavioural differences between sleep and wakefulness, the same resting‐state networks still support the falling asleep process. For instance, the DMN is preserved as during wakefulness (Deco, Hagmann, et al., 2014; Horovitz et al., 2008; Larson‐Prior et al., 2009), with observed increased activity changes in cortical areas at early N1 (Larson‐Prior et al., 2011; Picchioni et al., 2008). Similarly, an increase in BOLD signal fluctuation levels at the visual cortex was observed (Horovitz et al., 2008) with no evidence of reduced functional connectivity in sensory and association networks (Larson‐Prior et al., 2009). The dorsal attention network demonstrated a modest yet statistically significant increase in functional connectivity during light sleep (Larson‐Prior et al., 2009). Despite the maintenance of these networks during light sleep, as sleep deepens, functional connectivity transitions from a globally integrated state to smaller independent modules, exhibiting decreased long‐term temporal dependences (Boly et al., 2012; Spoormaker et al., 2012; Tagliazucchi et al., 2013). This is associated with the decreased conscious awareness and the brain's ability to integrate information. There is a gradual decrease in the connectivity of the frontoparietal regions, the posterior cingulate and retrosplenial cortices to the midposterior DMN node, and the contributions of the medial prefrontal cortex to the DMN (Sämann et al., 2011; Spoormaker et al., 2012). This occurs in a stepwise manner with increasing sleep depth, ultimately leading to the fragmentation of these connections, which sets the stage for subsequent sleep stages.

The transition to deep sleep is characterized by increased functional segregation (Madsen et al., 1991). This shift is consistent with changes in EEG delta power, suggesting a possible correlation between changes in brain network modularity and shifts in consciousness across sleep stages. Markers of reduced consciousness during deep sleep, such as preservation of posterior connectivity and decoupling of the medial prefrontal cortex, have been identified in studies (Horovitz et al., 2009; Koike et al., 2011; Sämann et al., 2011; Spoormaker et al., 2012). In addition, several studies have reported a decrease in DMN connectivity that correlates with the degree of consciousness impairment in minimally conscious, vegetative and comatose patients (Vanhaudenhuyse et al., 2010). Other reports of DMN reductions are documented by Blautzik et al. (2013) and Boveroux et al. (2010). Reduced activity in frontal areas is consistent with previous PET studies reporting decreased metabolism in these regions during N3 sleep, suggesting the presence of local slow‐wave activity (Stevner et al., 2019). Brain connectivity during deep sleep reveals a nuanced landscape of consciousness modulation, as evidenced by the distinctive patterns of brain connectivity and activity identified during different sleep stages. The intriguing paradox of diminished consciousness coexisting with increased activity in specific cortical regions challenges our understanding of the complexities underlying the brain mechanisms during sleep.

There is a lack of consensus regarding REM sleep and resting‐state networks connectivity. The connectivity of the DMN core regions appears to remain relatively stable across sleep stages, including REM sleep. Nevertheless, there is a notable reduction in the connectivity between the dorsomedial prefrontal cortex and the posterior cingulate cortex during REM sleep compared with NREM sleep. This reduction in frontoparietal connectivity is suggested to characterize REM sleep, with the ability to logically bind stored information significantly diminished due to dorsomedial prefrontal cortex dissociation, which may explain the prevalence of bizarreness in REM sleep dreams (Koike et al., 2011). Conversely, a reduction in DMN activity, occurring in synchrony with REMs, has been observed in the posterior cingulate and retrosplenial cortices (Hong et al., 2021), and fronto‐parietal and sensory‐motor networks have shown increases during REM sleep compared with decreased activity during SWS (Watanabe et al., 2014). Additionally, DMN hyperconnectivity during REM sleep was observed in a small sample of only two participants (Wu et al., 2012). In conclusion, the results of the studies reviewed indicate a complex connectivity pattern during REM sleep, with findings that are not entirely consistent with one another. A recent high‐density EEG study has demonstrated that both the breakdown and reconnection processes occurring during REM sleep are network‐ and frequency‐specific (Titone et al., 2024). This complexity, when considered alongside the challenges of acquiring REM sleep data inside the scanner, has resulted in under‐sampled studies. This highlights the necessity for increased efforts to investigate the neurocharacterization of REM sleep with reasonable sample sizes.

The thalamus serves as a gateway that regulates the flow of sensory inputs to the neocortex. It is highly connected to the cortex during wakefulness (Castro‐Alamancos, 2004). However, the thalamus disconnects from higher functional brain networks in the process of falling asleep, excluding thalamic nodes and highlighting increased functional connectivity between cortical regions (Spoormaker et al., 2010). This phenomenon was further supported by findings emphasizing altered thalamocortical functional connectivity during light sleep and its association with specific thalamic subdivisions and cortical projections (Andrade et al., 2011; Hale et al., 2016; Picchioni et al., 2014; Shmueli et al., 2007). These findings were also evidenced in fast‐fMRI (Setzer et al., 2022) and support the hypothesis that the thalamus plays a critical role in sleep–wake regulation (Jiang et al., 2021). The transition to deep sleep has been shown to result in a nuanced rearrangement of thalamic connectivity, this connectivity shift showed preserved propagation within the brainstem–thalamic axis and region‐specific effects in the cortex (Mitra et al., 2015). In summary, thalamic connectivity undergoes distinct patterns during different NREM sleep stages. The sleep onset shows a disconnection from higher brain networks, increased cortical connectivity, and specific thalamic and cortical associations. In contrast, the process of deep sleep involves a more intricate rearrangement of thalamic connectivity.

**FIGURE 1:**
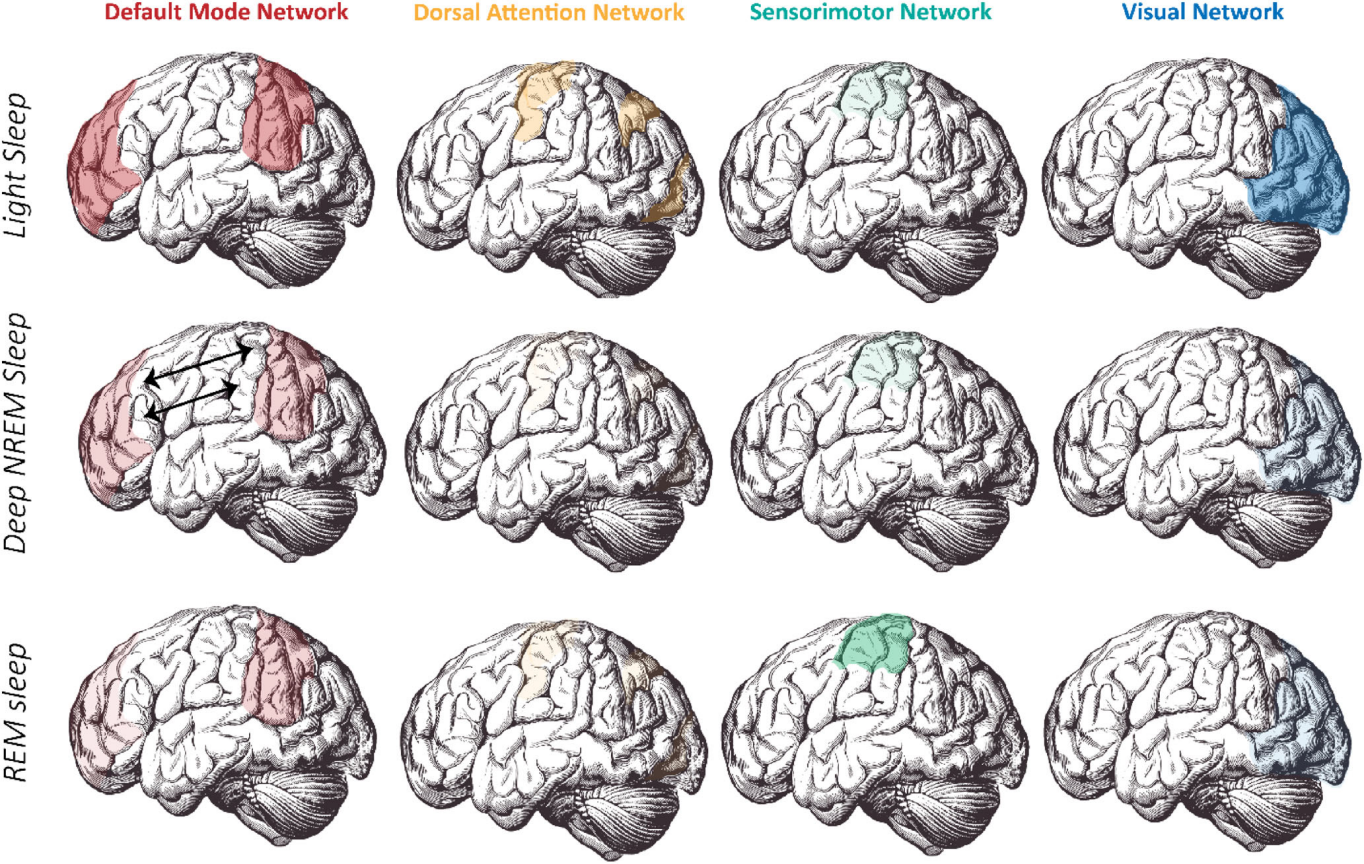
Functional connectivity patterns across different sleep stages. Light Sleep: the default mode network (DMN), which is characterized by brain activity without goal‐directed tasks, is preserved similarly to wakefulness, with increased connectivity in the dorsal attention network and heightened blood oxygenation level‐dependent (BOLD) signal fluctuations within the visual network. Deep non‐rapid eye movement (NREM) Sleep: DMN connectivity is significantly reduced, especially between the parietal cingulate cortex (PCC) and the medial prefrontal cortex, with the medial prefrontal cortex becoming decoupled from the rest of the DMN. Rapid eye movement (REM) Sleep: DMN activity is further reduced compared with deep NREM sleep, with decreased connectivity between the dorsomedial prefrontal cortex and the PCC. REMs‐locked DMN activity is reduced, while activity in the sensorimotor network is increased.

In summary, the study of neural activity during different stages of sleep reveals a complex and dynamic interplay between brain networks and consciousness. Resting‐state functional networks, such as the DMN, are crucial in shaping connectivity patterns during wakefulness and sleep stages. The DMN shows preserved connectivity during light sleep but changes during deep sleep, accompanied by a breakdown of long‐range functional connectivity. Thalamic connectivity also undergoes distinct patterns, with light sleep onset showing disconnection from higher brain networks and increased cortical connectivity, while deep sleep involves subtle rearrangements. The DMN is attenuated during REM sleep, suggesting deactivation during this phase. However, connectivity involving the inferior temporal gyrus to core DMN regions is more robust during REM sleep than during deep NREM sleep, suggesting higher or wake‐like brain activity during REM sleep (Figure 1). A caveat to REM sleep findings is that published studies have relied on small sample sizes due to the challenges of obtaining REM sleep in the scanner. More studies with larger sample sizes are needed to support or refute the current literature. As we unravel the complexities underlying brain mechanisms during sleep, these findings open new avenues for research and contribute to a broader perspective on the intricate relationship between brain networks, consciousness and sleep stages.

## 3 NEUROIMAGING SIGNATURES OF SLEEP MICROSTRUCTURE

This section will provide an overview of the findings in the neuroimaging studies of sleep microstructure. The term “event‐related” will be used to describe the correlation patterns in brain activity data during EEG‐defined sleep stages. This type of study involves simultaneous EEG and another neuroimaging modality such as fMRI. We will examine the relationship between specific sleep features such as vertex waves, spindles, K‐complexes and slow waves with brain activity data. Each sleep stage has its own unique features, and brain oscillations are crucial in defining each stage and may serve particular functions in the brain. In this section, we will summarize the findings in two areas: NREM sleep features (vertex waves, spindles, slow waves and K‐complexes); and REM sleep features (PGO waves and rapid‐eye‐movements).

### 3.1 Vertex waves

Vertex sharp transients have gained less attention despite their frequent occurrence and relevance for sleep onset. The specific EEG features of vertex waves comprise a large negative discharge with a particular waveform, narrower and more focal than K‐complexes. Vertex waves are thought to be a direct response to an external stimulus or a mechanism to sustain sleep after a stimulus. The first imaging study of the anatomical correlates of vertex sharp transients found regions of maximal local signal changes located at the paracentral cortex, medial occipital cortex, right and left superior temporal cortex, and right and left pre‐central cortex (Stern et al., 2011). The findings indicate that vertex waves, which are associated with brief multimodal sensory experiences and may modulate awareness of the external world during NREM sleep, are mainly localized at the primary sensorimotor cortices, a distribution that differs from that of sleep spindles. Therefore, it is suggested that vertex waves are not a gating of sensory function at a central location, such as the limbic system or the thalamus, but rather a distributed phenomenon in the neocortex that may be correlated to hypnagogic experiences taking place at the beginning of NREM sleep.

### 3.2 Spindles

Sleep spindles are a hallmark pattern of NREM sleep stage 2, and can be defined as a train of distinct waxing and waning waves with a frequency between 11 and 16 Hz (most commonly 12–14 Hz) with a duration of at least 0.5 s (Berry et al., 2012). Sleep spindles were observed in the first sleep recordings by Loomis et al. (1935), mostly occurring in N2 stage of the night and uncorrelated with heartbeat, respiration or muscle activity. Over the last decades, interest in understanding the function of sleep oscillations has increased considerably. Although the function of spindles is still unclear, several studies indicated its important role in memory consolidation and the relationship between certain features of spindles with age and intelligence (De Gennaro & Ferrara, 2003; Ujma, 2021). The latter might be explained by the fact that spindles to some extent highlight the efficiency of brain connectivity mechanisms needed to ensure efficient processing and integration of information, as shown by the relation between sleep spindles and white matter diffusion (Piantoni et al., 2013).

Based on the division criterion that slow spindles (< 13 Hz) predominate over frontal EEG derivations, and fast spindles (>13 Hz) over centroparietal derivations, Schabus et al. (2007) investigated the brain regions related to the two distinct types of sleep spindles, while Andrade et al. (2011) analysed the hippocampal‐neocortex connectivity of sleep spindle occurrence. These results showed the same origin in the thalamus for both spindles, but different activation patterns in the cortex. Both spindle types showed a common activation pattern in haemodynamic encompassing the anterior cingulate cortex, left anterior insula and superior temporal gyrus. But fast spindles expanded more broadly across the cortex, showing strong activations in the supplementary motor area, sensorimotor and mid‐cingulate cortex, whereas slow spindles correlated predominantly with activity in the right superior frontal gyrus. Caporro et al. (2012) also reported correlations with the posterior cingulate and right paracentral cortex; however, they only stated that these were central spindles, without specifying the frequency. These findings support the existence of two spindle types during human NREM sleep, and it has been suggested that fast spindles participate in the processing of sensorimotor and mnemonic information. Additionally, functional connectivity between the hippocampus and the neocortex exhibited a stable interaction with fast spindles, most pronounced in the subiculum, lateral temporal, insula, cingulate and medial prefrontal cortices (Andrade et al., 2011). However, no specific hippocampal activation was directly associated with slow or fast spindles. This suggests that spindle activity may increase functional connectivity between hippocampal and neocortical regions, but that it is not the only cause of connectivity. For more mechanisms and functions of spindles, see Fernandez's review paper (Fernandez & Lüthi, 2020).

### 3.3 Slow waves and K‐complexes

The NREM sleep is dominated by spindles and slow waves. The slow waves characterized by a frequency range of 0.5–2 Hz and peak‐to‐peak amplitude greater than 75 μV were first described in intracellular recordings obtained from anaesthetized cats. Slow waves can be observed in most cortical areas, especially in the primary sensory, association and motor cortices. However, the prevalence of slow waves in the primary visual cortex is lower (Steriade & McCarley, 2005). Tüshaus et al. (2017) further confirmed the role of the prefrontal cortex in slow wave generation. Frontal activation during slow‐wave activity, although no association with the thalamus, was also reported using PET by Dang‐Vu et al. (2005), in line with previous EEG studies (Finelli et al., 2001; Happe et al., 2002; Werth et al., 1997). Subsequent work has shown that the process of falling asleep can be characterized by large steep widespread slow waves, named type I slow waves, that are source‐localized to the medial prefrontal cortex and sensory‐motor areas and are thought to be generated in the brainstem. Once sleep deepens, type II slow waves are characteristically smaller and shallower, and are not originated in any specific cortical area (Bernardi et al., 2018; Siclari et al., 2014). How does the amplitude of slow waves reflect in fMRI‐assessed brain activity? Dang‐Vu et al. (2008) studied medium (75–140 μV) and high (>140 μV) amplitude slow waves, and the results indicated an association between activity in mesial‐temporal areas and slow‐wave amplitude, with medium‐amplitude waves preferentially activating frontal areas, and high‐amplitude waves being related to brainstem and para‐hippocampal activations. These findings suggested that different amplitudes are differently distributed across the scalp when compared with baseline activity. Specifically, higher neuronal synchronization results in larger amplitude of slow waves activating mesial‐temporal areas and possibly facilitating memory consolidation during NREM sleep.

K‐complexes are sparse occurrences of often large and isolated slow waves during N2 sleep, and are characterized by a brief positive wave followed by a larger negative wave and then by a positive wave again (Loomis et al., 1935). They are generated by the widespread occurrence of outward dendritic currents in cortical areas from the middle to upper layers of the cerebral cortex, usually accompanied by a decrease in EEG power, leading to reduced neural network activity (Cash et al., 2009). Caporro et al. (2012) investigated the fMRI of K‐complexes, finding the fMRI signal associated with K‐complexes comprises regions involved with spindles and vertex sharp transients, being maximal at the right post‐central gyrus, right pre‐central gyrus, left pre‐central gyrus, right thalamus, right insular cortex and right superior temporal gyrus. These findings contrasted with previous results by Laufs et al. (2007) that identified widespread signal decreases involving the thalamus, frontal, central, temporal and parts of the occipital cortices. However, both results are consistent with the cortical down‐state theory of K‐complexes (Cash et al., 2009). Jahnke et al. (2012) applied dynamic causal modelling (DCM) to fMRI data acquired during sleep to investigate the causal hierarchy associated with fMRI responses to K‐complexes. This study revealed that K‐complexes simultaneously inhibit arousals and allow passive processing of incoming sensory information.

Recently, Fultz et al. (2019) identified coupled electrophysiological, haemodynamic and cerebrospinal fluid (CSF) dynamics during NREM sleep. By acquiring fMRI data at high temporal resolution, the fast acquisition can also detect fluid inflow arriving at the edges of the imaging volume, thus allowing the authors to measure CSF flow dynamics simultaneously with the BOLD signal. First, they reported that CSF signal shows large oscillations (~0.05 Hz) during NREM sleep, while CSF small‐amplitude (~0.25 Hz) signal was observed during wakefulness. In addition, nearby non‐CSF regions did not exhibit such an effect. Next, they observed increases in BOLD signal amplitude in cortical grey matter regions compared with wakefulness, consistent with previous studies showing low‐frequency BOLD fluctuations during sleep. Additionally, the CSF signal was strongly temporally coupled to large fluctuations in the cortical grey matter BOLD signal during sleep, showing a strong anticorrelation that may indicate an alternation of blood flow and CSF flow during NREM sleep. To understand the potential mechanism, the authors hypothesized that EEG slow‐delta (0.2–4 Hz) oscillations might be coupled to blood volume oscillations, leading to changes in CSF flow. They found that neural oscillations preceded CSF oscillations, with a peak in EEG slow‐delta (0.2–4 Hz) oscillations occurring 6.4 s before the CSF peak. This work discovered that large waves of CSF flow appear during sleep, and identifies slow neural activity as a potential contributing mechanism to driving CSF flow.

Several researchers have found strong fMRI signal changes coinciding with K‐complexes, including the above study (Caporro et al., 2012; Fultz et al., 2019; Jahnke et al., 2012; Özbay et al., 2019). These may reflect the temporary decrease in neuronal activity during the cortical down state attributed to them (Cash et al., 2009). Fultz et al. (2019) found that these fMRI changes are associated with CSF pulsations and, therefore, that K‐complexes may have relevance for brain waste clearance through the glymphatic system, which has been shown to be more active during sleep (Xie et al., 2013). However, it is important to consider that large slow waves during N1 and N2 sleep (called type I slow waves; Bernardi et al., 2018; Siclari et al., 2014) are distinctly different than the type II slow waves that dominate N3. In fact, type I slow waves like K‐complexes are often accompanied by autonomic arousal (Colrain, 2005), while the latter have little autonomic correlate. Importantly, autonomic variability, including changes in heart rate and respiration, have been recognized as strong contributors to BOLD fMRI global signal (GS) fluctuations (Birn et al., 2006; Chang et al., 2009; Shmueli et al., 2007). To investigate the possible contribution of sleep‐specific autonomic contributions to CSF pulsations, recent work examined the lag between slow waves, GS reductions and CSF pulsations (Özbay et al., 2019; Picchioni et al., 2022), and considered both electrocortical and autonomic contributors. In the neural pathway, vasoconstrictions lag reductions in electrocortical activity by the well‐established 4–6 s delay dictated by the haemodynamic response. Autonomic pathway delays are longer and may reach 12–15 s, owing to the more sluggish effects of sympathetic and respiratory activity on vascular tone (Picchioni et al., 2022). Indeed, these researchers found the lag between slow‐wave activity and BOLD to average 13.7 s for the approximately 30 hr of N2 data considered. Thus, autonomic activity is an important contributor to CSF pulsations during N2 sleep. Data from this and future studies should be further analysed to quantify the relative contribution of autonomic effects. This does not take away the possibility that during N3, where slow‐wave activity is prevalent but not associated with autonomic arousals, neurovascular responses are the driving factor of CSF pulsations. However, because BOLD GS fluctuations (and accompanying CSF pulsations) are typically relatively small during N3 (e.g. see fig. 2 in Picchioni et al., 2022), simply the density of slow waves does not appear to be the determining factor in the generation of CSF pulsations. However, precisely how large‐scale CSF flow relates to clearance remains poorly understood. Intriguingly, a recent MRI study used a contrast agent injected into the CSF to directly measure brain waste clearance in humans, and showed that sleep induces faster clearance (Eide et al., 2021), highlighting the importance of understanding fluid transport during sleep. More research is needed to explore the relationship between slow‐wave activity and brain clearance (for review, see Lewis, 2021). As will be discussed below, these conclusions point to the importance of accounting for autonomic effects when interpreting EEG‐fMRI correlations, especially with arousal variations (Duyn et al., 2020; Özbay et al., 2019; Soon et al., 2021).

Much of this discussion does not consider the functional role of K‐complexes/type I slow waves and the associated neuroimaging activity in terms of waking cognitive outcomes. As Naji et al. (2019) showed, there is a positive correlation between overnight improvement in a declarative memory task and the extent that phasic increases in heart rate are time‐locked to 0.4–3.3 Hz waves during N2 and N3 sleep. This is aligned with prior work because, as reviewed by McGaugh (2013), sympathetic nervous system activity occurring subsequent to memory encoding still improves recall. This or similar ideas must be considered when designing future neuroimaging studies of K‐complexes/type I slow waves.

### 3.4 PGO waves and rapid‐eye‐movements

The PGO waves are described as phasic bioelectrical potentials occurring either in isolation or in bursts during the transition from SWS to REM sleep or even during REM sleep itself. PGO waves that trigger the bursts of rapid eye movements observed in REM sleep are mostly recorded in the pons (Jouvet, 1959), the lateral geniculate bodies (Mikiten, 1961) and the occipital cortex (Mouret et al., 1963), but can also be observed in other parts of the animal brain (Hobson, 1964). Among other functions, PGO waves during REM sleep are hypothesized to promote brain development and to facilitate brain plasticity (Gott et al., 2017). REMs during REM sleep are likely generated by similar PGO mechanisms in man as in animals. In humans, during REM sleep but not wakefulness, ocular movements density significantly correlated with rCBF in the mesencephalon and the thalamus, including the lateral geniculate body, the right parahippocampal gyrus, the striate cortex, the precuneus, the right anterior cingulate cortex and the supplementary motor area (Peigneux et al., 2001). Similar findings were reported using fMRI by Wehrle et al. (2005), who found activity in secondary cortical areas, basal ganglia, the cingulate midline attentional system and the midbrain. In the same line, Ioannides et al. (2009) took opportunity of the high temporal resolution of magnetoencephalographic (MEG) recordings to evidence that PGO activity bursts precede the onset of the rapid eye movement. Investigations of the visual cortices and their projections during REM sleep suggest a mechanism underlying REM sleep, where paralimbic projections of the visual cortices dissociate from the hierarchy of visual regions mediating perception of the external environment. Such a dissociation may explain some features of dreaming and the absence of reflective awareness (Braun et al., 1998).

The PET studies correlated the occurrence of REMs with cerebral blood flow in the visual cortex, thalamus, dorsolateral prefrontal cortex, anterior cingulate cortex, putamen, pons and amygdala (Hong et al., 1997; Peigneux et al., 2001). Using simultaneous fMRI and polysomnography recordings during REM sleep, Wehrle et al. (2005) found BOLD signal increases in the geniculate body and occipital cortex in close temporal relationship to REMs during human REM sleep. In subsequent studies, Miyauchi et al. (2009) not only confirmed that significant activation accompanying REMs in the lateral geniculate nucleus and the bilateral primary visual cortex, but also revealed that activation of the pontine tegmentum, ventroposterior thalamus and primary visual cortex started before REM onset, whereas activation of the putamen, anterior cingulate, parahippocampal gyrus and amygdala accompanied REMs using an event‐related analysis time‐locked to the occurrence of REMs. Moreover, as a control group, subjects made self‐paced saccades in total darkness showing no activation in the visual cortex. The above brain regions whose activity correlates with REMs were also confirmed by research by Hong et al. (2009), and those regions are similar to the brain structures involved in the generation of PGO waves, as previously reported in animal studies (Callaway et al., 1987), thus suggesting the presence of similar processes occurring during human REM sleep. Unexpectedly, Hong et al. (2009) showed REMs‐related activation also occurred in non‐visual sensory cortices, motor cortex, language areas and the ascending reticular activating system. One possible reason for their distributed REM‐locked activation is that instead of gold‐standard electrooculogram (EOG), they used video monitoring of eye movements that detected approximately four times as many REMs. In brief, these studies indicate a sharing mechanism beyond the expected visual scanning mechanisms between waking and dreaming. Regarding the studies conducted in REM sleep, it should be taken into account that whereas NREM sleep oscillations and phasic events (e.g. slow waves, spindles, K‐complexes) have been extensively studied and delineated, more studies are still needed to address with the same level of details the heterogeneous nature of REM sleep with its phasic and tonic constituents (Simor et al., 2020).

## 4 NEUROIMAGING CORRELATES OF SLEEP PHENOMENOLOGY

Neuroimaging techniques have provided valuable insights into the neural correlates of sleep stages and subjective sleep experiences such as dreaming and sensory processing. This section will review the neuroimaging findings on background activity during sleep and its relationship with sleep phenomenology. Specifically, we will explore the concurrent brain activity during dreaming and sensory processing during sleep, linking brain structural measures to sleep‐related behaviour outcomes, and the coupling between sleep features and brain structural measures. These subsections aim to provide a comprehensive overview of the neural underpinnings of sleep‐related phenomena and the implications for sleep‐related behaviour outcomes.

### 4.1 Background activity concurrently with dreaming and sensory processing during sleep

The investigation into brain activity during sleep has greatly advanced through the application of neuroimaging techniques. These methodologies have not only provided insights into the neural correlates of sleep stages, but have also offered valuable information on event‐related activity and subjective sleep experiences such as dreaming, lucid dreaming and sensory processing. This comprehensive understanding of background activity during sleep serves as a window into the underlying mechanisms of sleep and its various phenomena. In this section, we will review the neuroimaging findings on background activity during sleep and its relationship with sleep phenomenology.

To investigate the neural mechanisms underlying the content of dream experiences during REM sleep, Dresler et al. (2011) exploited the rare phenomenon of lucid dreaming, in which individuals become aware of their dream state and exhibit wake‐like cognitive abilities while in physiological REM sleep (Baird et al., 2019). Lucid dream experts were instructed to perform a sequence of left‐ and right‐hand movements, alternating with left–right–left–right eye movements, during lucid dreaming or while engaged in both an imagined and actual waking hand‐clenching task. The fMRI recordings during lucid REM dreams revealed increased BOLD signals in the sensorimotor cortex contralateral to the side of movement. In particular, activation during dreaming showed more localized patterns than during wakefulness, consisting of small clusters indicating either weaker or focal activation exclusively in hand areas. These findings marked the first demonstration of specific dream content during lucid dreaming, reinforcing that activation of motor imagery closely aligns with patterns associated with motor execution. Subsequently, Dresler et al. (2012) directly compared the neural correlates of lucid dreaming versus non‐lucid REM sleep using fMRI recordings from two stable lucid dreaming episodes. The study revealed increased activity in the right dorsolateral prefrontal cortex, consistent with previous EEG studies of lucid dreaming (Voss et al., 2009). The most pronounced activation occurred in the precuneus during lucid dreams as opposed to non‐lucid REM dreams. Interestingly, despite the usual impairment of working memory in ordinary dreams, the authors observed activation in the parietal lobules and activation in the dorsolateral prefrontal cortex, suggesting potential working memory demands. In addition, increased activation in bilateral frontopolar areas was noted, suggesting a possible link to the processing of internal states.

Is functional connectivity at the anterior prefrontal cortex associated with lucid dreaming frequency? Frequent lucid dreamers, compared with a control group, showed increased resting‐state functional connectivity between the left anterior prefrontal cortex and the bilateral angular gyrus, right inferior frontal gyrus and bilateral middle temporal gyrus (Baird et al., 2018). These findings, combined with the reported case study of lucid dreaming (Dresler et al., 2012), suggest that lucid dreaming frequency is associated with increased BOLD connectivity between the anterior prefrontal cortex and temporoparietal areas. The anterior prefrontal cortex and inferior parietal lobule/angular gyrus also exhibit reduced rCBF during REM sleep compared with wakefulness (Braun et al., 1997; Braun et al., 1998; Maquet et al., 1996). In addition, Eichenlaub et al. (2014) found that high dream recallers show higher rCBF in the temporoparietal junction and the medial prefrontal cortex during REM sleep and wakefulness compared with low dream recallers. These results suggest that the temporoparietal junction and the medial prefrontal cortex are involved in the dream recall process, and support the hypothesis of an association between lucid dreaming frequency and increased BOLD connectivity between the anterior prefrontal cortex and temporoparietal areas.

Another interesting topic of research is how the brain processes external stimuli during sleep. Although sleep is typically viewed as a state of behavioural unresponsiveness, it does not mean the brain is not receptive to external sensory inputs (Blume et al., 2018). In fact, a wide range of studies have shown that the primary sensory cortex can still be activated by external stimuli during sleep in adults (Portas et al., 2000; Wilf et al., 2016) and children (Redcay et al., 2007; Wilke et al., 2003). However, other studies have shown decreased activation of the sensory cortex when compared with wakefulness (Born et al., 2002; Czisch et al., 2002), with this decrease being linked to the presence of K‐complexes, thought to be a sleep protection mechanism (Czisch et al., 2004). Event‐related studies have also found that stimuli‐related brain activation during NREM sleep is correlated with the presence of sleep spindles or the phase of K‐complexes (Czisch et al., 2009; Dang‐Vu et al., 2011; Schabus et al., 2012). Using an acoustic oddball paradigm, Czisch et al. (2009) reported a prominent negative BOLD response for (rare) tones, yet no wake‐like activation of the auditory cortex. In their data, only rare tones, followed by an evoked K‐complex, were associated with a wake‐like activation of task‐related areas in the temporal cortex. Additionally, the phase of the K‐complex did not appear to alter brain responses in the thalamus and primary sensory cortex, but it does modulate the responses at higher cortical levels as shown in the superior temporal gyrus (Schabus et al., 2012). Moreover, sound‐related brain activations are constrained to the caudal part of the inferior colliculus when sounds are played during sleep spindles, whereas similar activations can occur in the auditory cortex when sounds are played in the absence of sleep spindles (Dang‐Vu et al., 2011). These studies supported the “Thalamic Gating Hypothesis”, which proposes that the thalamus acts as a gatekeeper during sleep and is mediated by spindles and K‐complexes that drive the activity of cortico‐thalamic loops (McCormick & Bal, 1994). These findings provide evidence that spindles and K‐complexes serve as sleep‐protective mechanisms while partially supporting the role of the thalamus as a gatekeeper during sleep.

### 4.2 Sleep features and brain structural coupling

Previous research has extensively investigated the correlation between brain activity and sleep characteristics. However, the relationship between brain structural measures and brain function during sleep remains under‐investigated. Tagliazucchi et al. (2016) explored the influence of anatomical connectivity on changes in functional connectivity between wakefulness and deep sleep. Their findings revealed regional differences, with primary sensory, motor, auditory and visual cortices showing increased structural–functional coupling during N2 and N3 sleep compared with wakefulness. In contrast, frontoparietal regions exhibited a disconnection between structure and function. Notably, coupling between structural and functional networks increased during deeper sleep NREM stages but not during light sleep (N1). These findings align with previous research indicating divergent cortical dynamics during NREM sleep and suggest a convergence of structural and functional connectivity near a critical point, facilitating efficient and controlled neural propagation (Deco, McIntosh, et al., 2014; Tagliazucchi et al., 2016).

Sleep spindles have been shown to have distinct features and can be characterized in terms of the frequency range, for instance slow (< 13 Hz) and fast (>13 Hz) spindles (Schabus et al., 2007). Investigating the relationship between fast and slow spindles and structural measures can shed light on their precise functions. Saletin et al. (2013) combined EEG sleep recordings with high‐resolution sMRI to reveal that grey matter volume in interoceptive and exteroceptive cortical regions correlates with slow sleep spindles. Additionally, grey matter volume in the bilateral hippocampus was associated with fast sleep spindles, supporting their role in declarative memory processing. Individual differences in slow‐wave oscillations, linked to grey matter volume in the basal forebrain and medial prefrontal cortex, further underscore the potential connection between sleep physiological phenomena and macroscopic brain structure. Another topic of interest is brain plasticity, i.e. the structural brain changes as a consequence of learning and post‐training sleep, probing the links between MR structural measurement‐related modifications and the underlying microstructural brain processes, and bidirectional influences between structural and functional brain changes (for review, see Stee & Peigneux, 2021).

White matter tracts constitute the brain's neuronal structural foundation, and alterations in neural activation may alter sleep spindles and slow‐wave oscillations. Based on this association, Piantoni et al. (2013) observed that higher spindle power correlated with higher D_a_ (axial diffusivity) in the forceps minor, anterior corpus callosum, temporal lobe areas and the thalamus. Individuals with a steeper rising slow‐wave slope showed higher D_a_ in the temporal fascicle and frontal white matter tracts. Consistent with these findings connecting white matter integrity as a predictor of quantitative and qualitative features of sleep spindles in young adults, Mander et al. (2017) showed that age‐related degeneration of white matter tracts is associated with reduced sleep spindles in older adults. Consequently, human brain white matter integrity influences sleep spindle decline in older adults, and thus sleep‐dependent motor memory consolidation in later life more than age per se.

### 4.3 Neuroanatomical correlates of sleep‐related behaviour outcomes

The influence of brain structure on behaviour is a central challenge in scientific research, with various statistical and mathematical models helping to identify significant relationships between brain structural metrics (e.g. cortical thickness, volume, microstructural estimates) and behavioural outcomes (e.g. questionnaires, task‐specific scores). A study associating dream recall frequency with cerebral blood flow at the medial prefrontal cortex and temporoparietal junction linked increased white matter density in the medial prefrontal cortex to high dream recallers, offering an anatomical counterpart to functional changes observed in previous studies. Vallat et al. (2018) compared grey and white matter measures between high and low dream recallers, and did not find significant differences in grey matter density between high and low recallers. However, increased white matter density in the medial prefrontal cortex was observed. This result introduces an anatomical counterpart to multiple findings reporting functional changes between high and low dream recallers. It also supports lesion studies that showed a cessation of dream reports after damage localized to the lateral ventricles' frontal horns (Solms, 1997). For frequent recallers of lucid dreams, Filevich et al. (2015) reported a higher grey matter volume in the frontopolar cortex compared with individuals with low lucid dreaming frequency. While Baird et al. (2018) were not able to replicate these structural findings, both studies reported functional differences related to the frontopolar cortex during wakefulness in high versus low lucid dream recallers.

In sleep research, DTI has been employed, for instance, to investigate associations of brain microstructural properties with sleep quality and duration. Khalsa et al. (2017) investigated changes in fractional anisotropy and mean diffusivity concerning these sleep variables. Sleep patterns were measured during 14 days using actigraphy and sleep diaries. The authors reported positive correlations between sleep duration and fractional anisotropy in the left orbitofrontal region and the right superior corona radiata. In contrast, sleep duration negatively correlated with mean diffusivity in right orbitofrontal white matter and the right inferior fasciculus. Moreover, sleep quality was associated with fractional anisotropy measures in the left caudate. Takeuchi et al. (2018) extended these findings in a cohort of over more than 1000 healthy young adults, revealing negative correlations between sleep quality and mean diffusivity in the prefrontal cortex and right hippocampus, while positive correlations between sleep duration and mean diffusivity were found in the prefrontal cortex and dopaminergic systems. These results suggest that total sleep time and subjective sleep quality are associated with subtle brain microstructural changes.

## 5 SLEEP NEUROIMAGING CHALLENGES AND FUTURE DIRECTIONS

**FIGURE 2:**
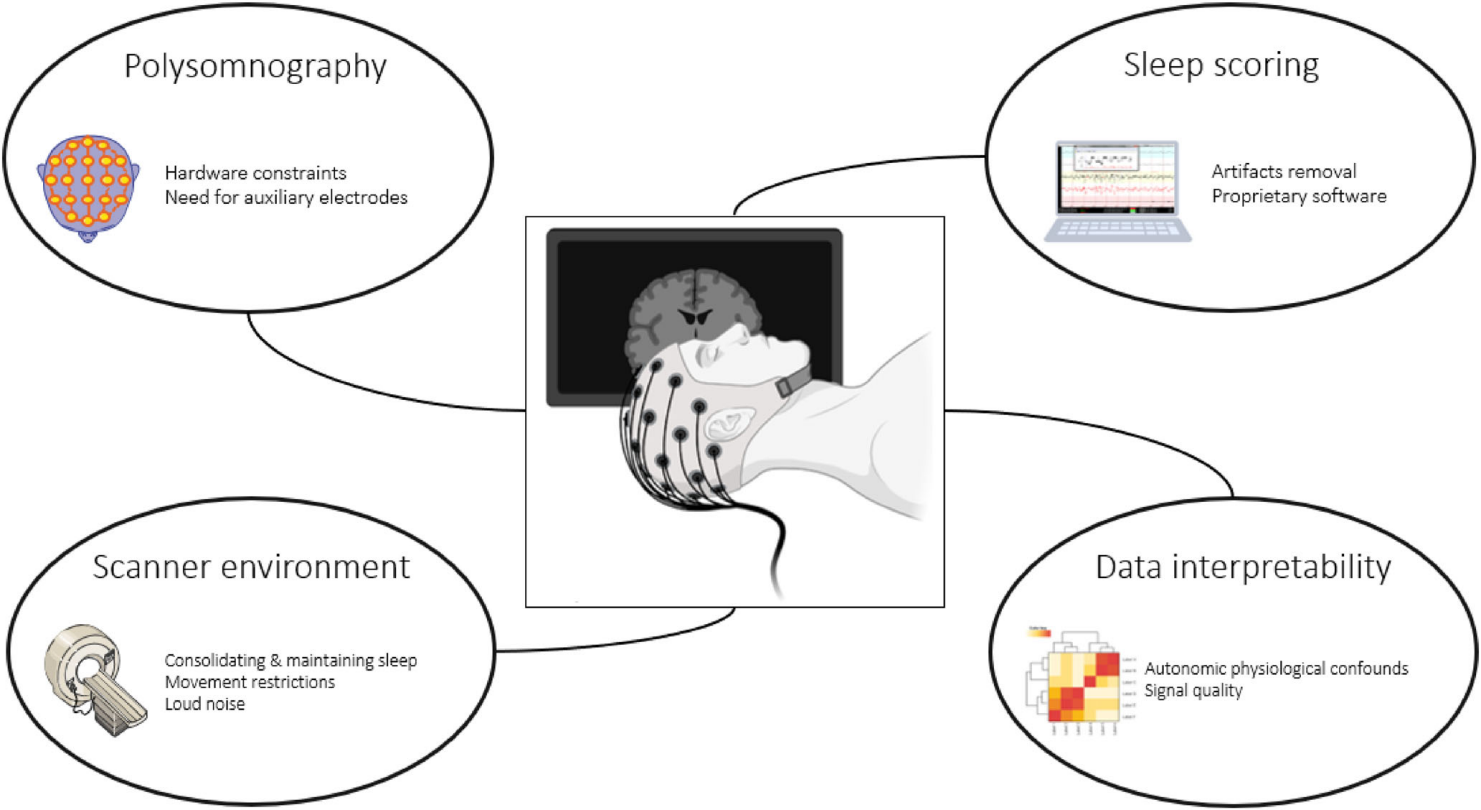
The most common challenges conducting sleep neuroimaging studies. Because polysomnography must be recorded to perform appropriate sleep scoring and identify electrophysiological microprocesses of interest such as sleep spindles or slow waves, hardware constraints might emerge, such as the limitation of adequate equipment, for instance, magnetic resonance imaging (MRI)‐compatible electroencephalography (EEG) caps and electrodes. Additionally, auxiliary electrodes and channels might be needed, which accounts for electrode placement, standardization and signal quality challenges. Sleep scoring online or offline becomes problematic, as data cleaning and artefact removal must be performed, particularly a concern for MRI studies. The lack of open‐source software does not facilitate individual‐based artefact removal algorithms, greatly benefiting sleep studies. Except for functional near‐infrared spectroscopy (fNIRS), any other scanner environment is restrictive, accounting for difficulties maintaining and consolidating sleep. Movement restrictions are a significant issue for MRI studies and can deteriorate the data due to movement artefacts. Acoustic noise in MRI could be reduced by developments and usability of silent MRI sequences combined with MRI‐compatible noise‐cancelling devices, such as headphones. Data quality and interpretability are crucial to advancing science. However, neuroimaging suffers from autonomic physiological confounds, especially during sleep. Current approaches usually model and regress physiological signals; however, it may account for signal loss. Developments in animal models and theoretical advances will help understand the complex relationship between metabolism, blood flow and neural activity.

Sleep neuroimaging comes with considerable challenges due to the unnatural environment that makes it difficult to consolidate and maintain sleep (Figure 2). The scanner setting requires movement restrictions, with the exception of NIRS, to avoid motion artefacts. The acoustic noise of MRI is not conducive to maintaining and consolidating sleep, and may affect its brain activity patterns. A further complication of sleep neuroimaging is the need to include polysomnography recordings that include recording brain activity (EEG), eye movements (EOG) and muscle activity (electromyography). These needs lead to additional hardware constraints, like the availability of auxiliary electrodes and channels, and the use of reference electrodes that may suffer from distortions and cardio‐ballistic artefacts (heart activity derived from electrodes placed near a pulsating vessel/artery) and are hard to correct. Furthermore, lengthy recordings cause discomfort to the subject, leading to difficulty maintaining sleep, EEG signal quality deterioration, and movement artefacts in the fMRI and EEG recordings, the latter through the induction of currents caused by the magnetic field. These limitations account for the high dropout rates in sleep studies compared with a standard task or resting‐state imaging during wakefulness. Moreover, it also limits the research questions the field can address. For instance, the homeostatic changes over the course of sleep have yet to be investigated, which requires long recording times.

Currently, sleep neuroimaging studies must use simultaneous physiological EEG recordings to perform sleep scoring accurately. From this perspective, we see two major challenges that we will address in terms of software and hardware advances. The first is difficulties in removing irregular artefacts from the EEG data, which is particularly challenging for MRI studies. Artefact removal software is complex and mostly designed by private companies with closed‐source code. Making such algorithms open‐source or partially accessible to the public would enable improvements in the field (Levitt et al., 2022), potentially leading to advances such as an adapted individual‐based artefact removal algorithm. Such a customized level would positively facilitate data pre‐processing without compromising the EEG signal in special cases where artefact removal implies data loss. Another possible way to facilitate artefact removal is the use of newly developed hardware. For instance, Chowdhury et al. (2014) developed a new EEG cap that incorporates embedded electrodes in a reference layer with similar conductivity to tissue and is electrically isolated from the scalp. In this new setup, the standard electrode layer is placed under the reference layer, which is in direct contact with the scalp, allowing the acquisition of mixed signals containing artefacts and neurophysiological signals. The reference layer electrodes are separated from the scalp, and only artefacts such as gradient artefacts, electrocardiogram and motion artefacts can be acquired. Therefore, the EEG signal without artefacts can be separated by comparing signals obtained from the standard and reference electrodes. Another solution might be integrating a carbon‐wired loop that has outperformed post‐processing EEG/fMRI artefact corrections (van der Meer et al., 2016). This method makes use of carbon‐wired loops as additional sensors that track both helium‐pump and cardio‐ballistic artefacts. Another promising direction is to develop sleep staging algorithms based on electrocardiogram or respiratory signals, as electrocardiogram presents a higher signal‐to‐noise ratio than EEG signal, and wearable devices measuring respiratory signals are already available in the market (Sun et al., 2020). The development of MRI‐based eye‐tracking can assist in the detection of eye movement positions during REM sleep, particularly interesting in lucid dreaming (Frey et al., 2021). Additionally, wearable and contactless devices could potentially help the field and decrease the experimental setup complexity.

To ensure optimal sleep stability in this unusual environment, the application of total or light sleep deprivation protocols is applied, thus ensuring increased sleep pressure leading to shorter sleep latency. However, sleep‐deprived subjects may account for potential confounds in homeostatic sleep regulation and impaired coupling of the DMN, among other physiological changes (Wang et al., 2020). In a recent study, Moehlman et al. (2019) confirmed a procedure to obtain all‐night fMRI data in sleeping subjects without sleep deprivation. The key detail was to perform acquisitions in consecutive nights, hence the first night served as an adaptation night, eliminating the need for systematic sleep deprivation. Although the authors acknowledged that the subjects were slightly sleep‐deprived after the first night (which may lead to sleep alterations on the second night), a washout period between the 2 nights might contribute to overcoming changes in sleep architecture due to sleep deprivation, while preserving the stability of sleep in the scanner. Besides having a consecutive‐nights experiment design, researchers should also consider using sleep hygiene protocols to enhance stable sleep under experimental conditions. For instance, maintaining a regular sleep routine, preferably overlapping with the experiment design, avoiding daytime naps, screen‐light and caffeinated beverages before bedtime can improve sleep quality and enhance the chances of falling asleep.

Movement restrictions during scanning are a critical restriction in sleep neuroimaging studies: both PET and MRI do not allow subjects to change positions, creating discomfort when measuring sleep, in particular during longer scanning periods. Additionally, PET imaging requires restricting one arm's movement as a catheter must be placed during scanning. Ongoing developments in the field may lead to flexible MRI apparatus or even portable scanners (Cooley et al., 2021; Corea et al., 2016). Recent developments in wearable MEG based on optically pumped magnetometers (Boto et al., 2018) have granted this electrophysiological imaging method a considerable advantage in comparison to neuroimaging modalities relying on rigid scanners such as MRI or PET. However, sleeping in different positions seems to cause alteration in brain activity patterns. In supine posture, the brain activities in the left precuneus and anterior cingulate cortex were greater than those in lateral positions (Xu et al., 2021). Once a flexible apparatus becomes a reality, more research is needed to investigate posture influence in brain activity and how upcoming research can correlate its findings with current literature.

The acoustic noise produced by MRI is not conducive to sleep and may affect brain activity during sleep. Silent sequences have been developed and applied, especially in acoustic stimulation tasks, for many years (Liebig et al., 2019; Lövblad et al., 1999; Schmitter et al., 2008). Since all‐night sleep fMRI studies became feasible, silent sequences in combination with noise cancellation systems are welcome allies in noise reduction, thus diminishing subject discomfort and enhancing sleep maintenance mostly with the drawback of reduced spatial resolution. This is critical as studies have shown that REM sleep can be particularly suppressed by acoustic noise and drastic environmental changes, which accounted for fewer neuroimaging studies on REM than NREM sleep (Mulert & Lemieux, 2009). However, noise cancellation headphones can also cause EEG artefacts, and therefore should be used with care. Researchers should consider the limitations imposed by the scanner environment when planning their studies, especially the timeline required to acquire reasonable sample sizes and the methods to study the sleeping brain, for instance, seed‐region, independent networks with component analysis, DCM, and graph theoretical analysis.

Among the most critical challenges for neuroimaging techniques are interpretability and signal quality. Because multiple neurophysiological and autonomic changes are correlated with neural activity, which fluctuates along the wake–sleep cycle, untangling these neural sources from their confounding consequences (e.g. changes in blood flow) is a complex challenge that cannot be overcome by recording and regressing physiological signals. Hence, one significant gap that needs to be addressed is how changes in autonomic physiology during sleep affect blood flow signals. Current approaches for minimizing effects contributing to the overall signal involve regressing out from the fMRI time‐series signals that reflect the effects one wishes to remove, for instance, GS, signals reflecting fluctuations in heart rate or respiration, or reference signal from regions as the white matter of CSF. However, depending on the study's goal, systemic effects may covary with neuronal effects, which might be partially excluded with the removal approach. This is crucial, especially for sleep studies, as cortical activity changes and systemic physiology may be derived from arousal state changes. Conversely, physiological changes may be triggered by neuronal activity. A good example is a study by Özbay et al. (2019) that analysed the temporal relationship between amplitude variations of vascular tone derived from photoplethysmography signal and the occurrence of EEG K‐complexes. They observed that fMRI signal showed clear covariations with EEG K‐complexes and vascular tone. More importantly, arousal changes lead to joint changes in cortical and autonomic activity (Özbay et al., 2019). These signal changes are related to shifts in autonomic and central nervous systems, emphasizing the importance of such contributions often neglected as noise when interpreting fMRI data. The autonomic system is also regulated by the central nervous system via the brainstem, which is a primary control centre of sleep and arousal regulation, and ties the common changes in electrocortical and autonomic activity that are so pronounced across the sleep–wake cycle (Duyn et al., 2020). These findings, also shown by Soon et al. (2021), emphasize the importance of modelling autonomic and neuromodulatory effects as these effects vary with the sleep stage, thus making comparisons of functional connectivity patterns across sleep states difficult. Importantly, the altered amplitude of BOLD signal fluctuations during sleep could modulate connectivity estimates even in the absence of any true change in correlation strength, due to the large change in signal amplitude. It seems unlikely that these challenges can be overcome without extensive animal studies and new theoretical insights on the relationship between metabolism, blood oxygenation and neural activity. Future experiments should go beyond temporal averages and try to find the time‐resolved signatures of different patterns of electrophysiological activity. Which local field potential (LFP) frequency bands contribute most to the signal acquired by different neuroimaging techniques? Can these methods pick up information beyond the characteristics of LFP oscillations, such as complexity? Is it possible to find a one‐to‐one relationship between electrophysiological activity parameters and the data provided by neuroimaging methods? Without advancing answers to these questions, the interpretability of neuroimaging data is very problematic. The assessment of signal quality depends on disentangling the contribution of neural activity from recording physiological and movement artefacts, and therefore relates to the challenges concerning interpretability. Computational models could be helpful to encode theoretical knowledge on the mapping between neuroimaging and LFP data, allowing to transcend what is directly available from empirical data. Still, it is possible that we are reaching a limit about the amount of neural information that can be decoded from standard neuroimaging data. Moving towards high‐resolution modalities (e.g. layer BOLD fMRI) could be necessary to push the borders of our knowledge. However, these advances alone will not solve the autonomic confound. Besides, higher field strength, such as 7 T, allows better resolution and sensitivity, but it compromises EEG signal quality. Hence, further development of EEG systems that can be used in higher field strength and suppress cardio‐ballistic and gradient artefacts should be explored.

The BOLD contrast results from various physiological variables, including blood flow and volume, local vascular architecture, cerebral oxygenation metabolic rate and autonomic processes. Unlike BOLD signal, perfusion fMRI provides non‐invasive and absolute quantification of cerebral blood flow analogously to PET scanning, utilizing standard MRI hardware and not requiring radioactive tracer administration (Detre et al., 2009). Perfusion techniques applied to fMRI are less sensitive to baseline shifts and do not rely on an imbalance between flow and oxygen consumption. Perfusion fMRI, such as arterial spin labelling (ASL), provides more of an absolute measure than BOLD, thus providing the opportunity to compare brain function without conventional task‐correlated BOLD fMRI directly. For instance, a predictive model is needed to perform the analysis: hand clenching or eye signals during REM lucidity. ASL fMRI has been applied to sleep studies with promising results during sleep (Tüshaus et al., 2017), although some disadvantages must be considered in perfusion studies regarding brain coverage and signal–noise ratio. Perfusion fMRI has a low temporal and spatial resolution, and adding proper quantitation capability reduces its sensitivity and is cumbersome. Furthermore, as a haemodynamic signal, many of the same interpretation problems are still there, and more studies are needed to test its feasibility for sleep research.

In summary, advances in neuroimaging have significantly improved our understanding of brain activity during sleep beyond traditional polysomnography‐based approaches. For instance, in Sections 2 and 3 of this review, we discussed in detail how early PET studies identified regional activations and deactivations across sleep stages, while newer techniques such as EEG/fMRI allow detailed characterization of transient sleep oscillations and neural processes within these stages. Functional neuroimaging research has revealed that the brain retains its capacity to respond to external auditory stimuli during sleep, indicating that certain aspects of information processing remain active. Additionally, spontaneous reactivation of brain regions associated with learning has been observed during sleep, and studies triggering reactivation using contextual cues during sleep further support the idea that neuronal replay and reactivation play a causal role in memory consolidation (for review, see Farthouat and Peigneux, 2015). Sleep and wakefulness are now widely recognized to occur and be regulated locally. Multimodal imaging techniques, which allow for the simultaneous tracking of global and local brain states, may contribute to our understanding of these local phenomena (Song & Tagliazucchi, 2020). In particular, spontaneous oscillations in fMRI BOLD activity, observed across both cortical and subcortical regions, have been proposed as potential markers of local sleep. These oscillations, which are detectable at the level of individual neuronal populations, may reflect the intensity of local sleep and offer valuable insights into monitoring local neuronal states and identifying the brain regions that first transition into or out of sleep during wake–sleep transitions (Song et al., 2022). Clinically, recent studies show that low‐frequency oscillations during sleep promote CSF dynamics, which aids in metabolic waste clearance (Fultz et al., 2019). This process is critical for clearing accumulated protein, such as amyloid beta and tau, associated with Alzheimer's disease, and sleep disturbances may reduce CSF flow and clearance efficiency, potentially worsening memory impairment and disease progression. These findings point to potential biomarkers for diagnosing and managing conditions related to impaired sleep or clearance mechanisms, linking neural activity, CSF dynamics and cognitive health. Additionally, data‐driven methods such as Hidden Markov Models (HMM) combined with EEG/fMRI recordings offer a more in‐depth understanding of brain states during sleep. Unlike arbitrary polysomnography‐based sleep staging, which segments sleep into fixed 30‐s epochs, HMM identifies temporally precise brain states and their transitions, revealing previously unobservable dynamics. Modular analyses of HMM states have identified distinct substates within NREM and REM sleep that correspond to polysomnography‐defined stages while revealing new patterns, such as the duality between phasic and tonic REM (Stevner et al., 2019; Yang et al., 2024). These methods emphasize sleep's dynamic nature, highlighting the importance of using advanced multimodal imaging techniques to enhance our understanding of the relationship between sleep physiological mechanisms and their contributions to restorative and memory processes.

## 6 CONCLUSIONS

This review summarized neuroimaging approaches to sleep research in healthy and non‐sleep‐deprived populations. Different neuroimaging modalities, when combined with electrophysiological recordings, have helped to bridge animal and human research by measuring in vivo functional and metabolic information with good spatial and temporal resolution. The advance of novel techniques has increasingly facilitated consecutive all‐night imaging recordings, perhaps the final technical challenge of sleep neuroimaging. The field has grown considerably from early findings contrasting wakefulness versus sleep measurements towards the analysis of fine‐grained sleep‐related events and the study of whole‐brain functional coupling across the human wake–sleep cycle. The combination of neuroimaging methods and new experimental protocols is further extending our current knowledge of poorly understood phenomena such as lucid dreaming and local sleep. Neuroimaging has also improved the interpretation of sleep disorders, has demonstrated the importance of sleep for different cognitive functions, particularly memory consolidation and learning, and has raised concerns regarding the severe consequences of sleep deprivation. Despite the significant advances brought by neuroimaging to the field of sleep research, much room for future investigation remains, especially concerning the replication of initial findings and the study of REM sleep, which is especially difficult to capture in the environment of an MRI scanner. Although many studies yielded valuable discoveries, small samples can lead to significant variability and potentially limit the reliability of conclusions drawn about sleep neuroimaging across different demographics or clinical populations. Future studies should prioritize larger, multisite studies and collaborations to improve statistical power and ensure findings are more broadly applicable. Other interesting open questions in the field are investigating the impact of wake intrusions during sleep and how inter‐individual and inter‐regional differences play a role in local sleep, as well as examining the influence of circadian rhythms on this phenomenon. Finally, the functions and mechanisms underlying dreaming remain unknown, thus future research should focus on investigating brain changes during lucid and non‐lucid REM sleep dreams and, in collaboration with thoughtfully‐designed dream interviews, uncover the differences that exist between NREM and REM dreams.

## AUTHOR CONTRIBUTIONS

**Mariana Pereira:** Conceptualization; writing – original draft; writing – review and editing; investigation. **Xinyuan Chen:** Writing – review and editing; investigation. **Anastasiya Paltarzhytskaya:** Investigation; writing – review and editing. **Yibran Pacheсo:** Investigation; writing – review and editing. **Nils Muller:** Writing – review and editing. **Leonore Bovy:** Writing – review and editing. **Xu Lei:** Writing – review and editing. **Wei Chen:** Writing – review and editing. **Haoran Ren:** Writing – review and editing. **Chen Song:** Writing – review and editing. **Laura D. Lewis:** Writing – review and editing. **Thien Thanh Dang‐Vu:** Writing – review and editing. **Michael Czisch:** Writing – review and editing. **Dante Picchioni:** Writing – review and editing. **Jeff Duyn:** Writing – review and editing. **Philippe Peigneux:** Writing – review and editing. **Enzo Tagliazucchi:** Writing – review and editing. **Martin Dresler:** Writing – review and editing; conceptualization.

## FUNDING INFORMATION

MP and MD were supported by a Vidi grant from the Dutch Research Council (NWO) and a research grant from the Bial Foundation; XC and XL were supported by grants from National Nature Science Foundation of China (31971028), and Major Project of Medicine Science and Technology of PLA (AWS17J012); CS is supported by the Wellcome Trust (209192/Z/17/Z); LL is supported by NIH R01‐AG070135; TDV LL is supported by NIH R01‐AG070135; TDV is supported by the Canadian Institutes of Health Research (grants MOP 142191, PJT 153115, PJT 156125 and PJT 166167), the Natural Sciences and Engineering Research Council of Canada, the Canada Foundation for Innovation and the Fonds de Recherche du Québec – Santé.

## CONFLICT OF INTEREST STATEMENT

All authors declare that they have no conflicts of interest to disclose for this paper.

## Data Availability

Data sharing not applicable to this article as no datasets were generated or analysed during the current study.

## APPENDIX A

**TABLE A1:** Summary of all discussed papers grouped per imaging modality and sleep‐specific topics.

	PET	fMRI	sMRI/DTI
Wake–NREM	Buchsbaum et al. (1989), Maquet et al. (1990), Braun et al. (1997), Andersson et al. (1998)	Horovitz et al. (2008), Horovitz et al. (2009), Picchioni et al. (2008), Picchioni et al. (2014), Koike et al. (2011), Larson‐Prior et al. (2009), Larson‐Prior et al. (2011), Sämann et al. (2011), Spoormaker et al. (2010), Spoormaker et al. (2012), Tagliazucchi et al. (2013), Boly et al. (2012), Wu et al. (2012), Deco et al. (2014), Hale et al. (2016), Mitra et al. (2015), Stevner et al. (2019), Jiang et al. (2021)	Tagliazucchi et al. (2016)
Light–deep NREM	Maquet et al. (1992), Kajimura et al. (1999), Madsen et al. (1991)	Koike et al. (2011), Sämann et al. (2011), Spoormaker et al. (2010), Tagliazucchi et al. (2012), (2013), (2014), Houldin et al. (2019), Stevner et al. (2019), Jiang et al. (2021)	
Wake–REM	Buchsbaum et al. (1989), Maquet et al. (1990) Maquet et al. (1996), Braun et al. (1997), Braun et al. (1998)	Houldin et al. (2019), Koike et al. (2011)	
NREM–REM	Braun et al. (1997), Braun et al. (1998)	Houldin et al. (2019), Koike et al. (2011)	
Vertex‐waves		Stern et al. (2011)	
Spindles	Hofle et al. (1997)	Schabus et al. (2007), Andrade et al. (2011), Caporro et al. (2012)	Saletin et al. (2013), Piantoni et al. (2013), Mander et al. (2017)
K‐complexes		Laufs et al. (2007), Caporro et al. (2012), Jahnke et al. (2012)	
Slow‐waves	Hofle et al. (1997), Tüshaus et al. (2017), Dang‐Vu et al. (2005)	Dang‐Vu et al. (2008), Picchioni et al. (2022), Xu et al. (2020), Fultz et al. (2019) (slow delta)	Saletin et al. (2013), Piantoni et al. (2013)
REMs	Hong et al. (1997), Peigneux et al. (2001)	Wehrle et al. (2005), Miyauchi et al. (2009), Hong et al. (2009)	
PGO waves	Peigneux et al. (2001)	Wehrle et al. (2005)	
Dreaming	Eichenlaub et al. (2014)	Dresler et al. (2011), Dresler et al. (2012)	Vallat et al. (2018), Filevich et al. (2015), Baird et al. (2018)
Sleep duration and quality			Khalsa et al. (2017), Takeuchi et al. (2018)
External stimuli		Portas et al. (2000), Born et al. (2002), Wilke et al. (2003), Redcay et al. (2007), Czisch et al. (2002), Czisch et al. (2004), Czisch et al. (2009), Dang‐Vu et al. (2011), Schabus et al. (2012), Wilf et al. (2016), Blume et al. (2018)	
Connectivity		Horovitz et al. (2008), Horovitz et al. (2009), Picchioni et al. (2008), Picchioni et al. (2014), Koike et al. (2011), Larson‐Prior et al. (2009), Larson‐Prior et al. (2011), Sämann et al. (2011), Spoormaker et al. (2010), Spoormaker et al. (2012), Tagliazucchi et al. (2012), (2013), Boly et al. (2012), Hale et al. (2016), Mitra et al. (2015), Houldin et al. (2019), Stevner et al. (2019), Jiang et al. (2021)	

Abbreviations: DTI, diffusion tensor imaging; fMRI, functional magnetic resonance imaging; NREM, non‐rapid eye movement; PET, positron emission tomography; PGO, ponto‐geniculo‐occipital; REM, rapid eye movement; sMRI, structural magnetic resonance imaging.

## References

[jsr14462-bib-0001] Alexander, A. L. , Lee, J. E. , Lazar, M. , & Field, A. S. (2007). Diffusion tensor imaging of the brain. Neurotherapeutics, 4(3), 316–329. 17599699 10.1016/j.nurt.2007.05.011 PMC2041910

[jsr14462-bib-0002] Andersson, J. L. R. , Onoe, H. , Hetta, J. , Lidström, K. , Valind, S. , Lilja, A. , Sundin, A. , Fasth, K. J. , Westerberg, G. , Broman, J. E. , Watanabe, Y. , & Långström, B. (1998). Brain networks affected by synchronized sleep visualized by positron emission tomography. Journal of Cerebral Blood Flow and Metabolism, 18(7), 701–715. 10.1097/00004647-199807000-00001 9663500

[jsr14462-bib-0003] Andrade, K. C. , Spoormaker, V. I. , Dresler, M. , Wehrle, R. , Holsboer, F. , Sämann, P. G. , & Czisch, M. (2011). Sleep spindles and hippocampal functional connectivity in human NREM sleep. Journal of Neuroscience, 31(28), 10331–10339. 10.1523/JNEUROSCI.5660-10.2011 21753010 PMC6623055

[jsr14462-bib-0004] Assaf, Y. , & Basser, P. J. (2005). Composite hindered and restricted model of diffusion (CHARMED) MR imaging of the human brain. NeuroImage, 27(1), 48–58. 15979342 10.1016/j.neuroimage.2005.03.042

[jsr14462-bib-0005] Baird, B. , Castelnovo, A. , Gosseries, O. , & Tononi, G. (2018). Frequent lucid dreaming associated with increased functional connectivity between frontopolar cortex and temporoparietal association areas. Scientific Reports, 8(1) 17798. 10.1038/s41598-018-36190-w PMC6290891 30542052

[jsr14462-bib-0006] Baird, B. , Mota‐Rolim, S. A. , & Dresler, M. (2019). The cognitive neuroscience of lucid dreaming. Neuroscience & Biobehavioral Reviews, 100, 305–323. 30880167 10.1016/j.neubiorev.2019.03.008 PMC6451677

[jsr14462-bib-0007] Bernardi, G. , Siclari, F. , Handjaras, G. , Riedner, B. A. , & Tononi, G. (2018). Local and widespread slow waves in stable NREM sleep: Evidence for distinct regulation mechanisms. Frontiers in Human Neuroscience, 12, 1248 Accessed October 22, 2022. 10.3389/fnhum.2018.00248 PMC6018150 29970995

[jsr14462-bib-0008] Berry, R. B. , Brooks, R. , Gamaldo, C. E. , Harding, S. M. , Marcus, C. , & Vaughn, B. V. (2012). The AASM manual for the scoring of sleep and associated events. Rules, Terminology and Technical Specifications, Darien, Illinois, American Academy of Sleep Medicine, 176, 2012.

[jsr14462-bib-0009] Birn, R. M. , Diamond, J. B. , Smith, M. A. , & Bandettini, P. A. (2006). Separating respiratory‐variation‐related fluctuations from neuronal‐activity‐related fluctuations in fMRI. NeuroImage, 31(4), 1536–1548. 10.1016/j.neuroimage.2006.02.048 16632379

[jsr14462-bib-0010] Blautzik, J. , Keeser, D. , Berman, A. , Paolini, M. , Kirsch, V. , Mueller, S. , Coates, U. , Reiser, M. , Teipel, S. J. , & Meindl, T. (2013). Long‐term test‐retest reliability of resting‐state networks in healthy elderly subjects and with amnestic mild cognitive impairment patients. Journal of Alzheimer's Disease, 34(3), 741–754. 10.3233/jad-111970 23271315

[jsr14462-bib-0011] Blume, C. , Del Giudice, R. , Wislowska, M. , Heib, D. P. , & Schabus, M. (2018). Standing sentinel during human sleep: Continued evaluation of environmental stimuli in the absence of consciousness. NeuroImage, 178, 638–648. 29859261 10.1016/j.neuroimage.2018.05.056

[jsr14462-bib-0012] Boly, M. , Perlbarg, V. , Marrelec, G. , Schabus, M. , Laureys, S. , Doyon, J. , Pélégrini‐Issac, M. , Maquet, P. , & Benali, H. (2012). Hierarchical clustering of brain activity during human nonrapid eye movement sleep. Proceedings of the National Academy of Sciences of the United States of America, 109(15), 5856–5861. 10.1073/pnas.1111133109 22451917 PMC3326471

[jsr14462-bib-0013] Born, A. P. , Law, I. , Lund, T. E. , Rostrup, E. , Hanson, L. G. , Wildschiødtz, G. , Lou, H. C. , & Paulson, O. B. (2002). Cortical deactivation induced by visual stimulation in human slow‐wave sleep. NeuroImage, 17(3), 1325–1335. 12414272 10.1006/nimg.2002.1249

[jsr14462-bib-0014] Boto, E. , Holmes, N. , Leggett, J. , Roberts, G. , Shah, V. , Meyer, S. S. , Muñoz, L. D. , Mullinger, K. J. , Tierney, T. M. , Bestmann, S. , Barnes, G. R. , Bowtell, R. , & Brookes, M. J. (2018). Moving magnetoencephalography towards real‐world applications with a wearable system. Nature, 555(7698), 657–661. 10.1038/nature26147 29562238 PMC6063354

[jsr14462-bib-0015] Boveroux, P. , Vanhaudenhuyse, A. , Bruno, M. A. , Noirhomme, Q. , Lauwick, S. , Luxen, A. , Degueldre, C. , Plenevaux, A. , Schnakers, C. , Phillips, C. , Brichant, J. F. , Bonhomme, V. , Maquet, P. , Greicius, M. D. , Laureys, S. , & Boly, M. (2010). Breakdown of within‐ and between‐network resting state functional magnetic resonance imaging connectivity during propofol‐induced loss of consciousness. Anesthesiology, 113(5), 1038–1053. 10.1097/ALN.0b013e3181f697f5 20885292

[jsr14462-bib-0016] Braun, A. R. , Balkin, T. , Wesenten, N. , Carson, R. E. , Varga, M. , Baldwin, P. , Selbie, S. , Belenky, G. , & Herscovitch, P. (1997). Regional cerebral blood flow throughout the sleep‐wake cycle. An H2 (15) O PET study. Brain: A Journal of Neurology, 120(7), 1173–1197. 9236630 10.1093/brain/120.7.1173

[jsr14462-bib-0017] Braun, A. R. , Balkin, T. J. , Wesensten, N. J. , Gwadry, F. , Carson, R. E. , Varga, M. , Baldwin, P. , Belenky, G. , & Herscovitch, P. (1998). Dissociated pattern of activity in visual cortices and their projections during human rapid eye movement sleep. Science, 279(5347), 91–95. 9417032 10.1126/science.279.5347.91

[jsr14462-bib-0018] Buchsbaum, M. S. , Gillin, J. C. , Wu, J. , Wu, J. , Hazlett, E. , Sicotte, N. , Dupont, R. M. , & Bunney, W. E., Jr. (1989). Regional cerebral glucose metabolic rate in human sleep assessed by positron emission tomography. Life Sciences, 45(15), 1349–1356. 10.1016/0024-3205(89)90021-0 2796606

[jsr14462-bib-0019] Callaway, C. W. , Lydic, R. , Baghdoyan, H. A. , & Hobson, J. A. (1987). Pontogeniculooccipital waves: Spontaneous visual system activity during rapid eye movement sleep. Cellular and Molecular Neurobiology, 7(2), 105–149. 3308096 10.1007/BF00711551 PMC11567225

[jsr14462-bib-0020] Campbell, J. S. W. , & Pike, G. B. (2019). Diffusion magnetic resonance imaging. 505–518.

[jsr14462-bib-0021] Caporro, M. , Haneef, Z. , Yeh, H. J. , Lenartowicz, A. , Buttinelli, C. , Parvizi, J. , & Stern, J. M. (2012). Functional MRI of sleep spindles and K‐complexes. Clinical Neurophysiology, 123(2), 303–309. 10.1016/j.clinph.2011.06.018 21775199 PMC3208090

[jsr14462-bib-0022] Carskadon, M. A. , & Dement, W. C. (2005). Normal human sleep: an overview. Principles and Practice of Sleep Medicine, 4(1), 13–23.

[jsr14462-bib-0023] Cash, S. S. , Halgren, E. , Dehghani, N. , Rossetti, A. O. , Thesen, T. , Wang, C. M. , Devinsky, O. , Kuzniecky, R. , Doyle, W. , Madsen, J. R. , Bromfield, E. , Erőss, L. , Halász, P. , Karmos, G. , Csercsa, R. , Wittner, L. , & Ulbert, I. (2009). The human K‐complex represents an isolated cortical Down‐state. Science, 324(5930), 1084–1087. 10.1126/science.1169626 19461004 PMC3715654

[jsr14462-bib-0024] Castro‐Alamancos, M. A. (2004). Dynamics of sensory thalamocortical synaptic networks during information processing states. Progress in Neurobiology, 74(4), 213–247. 10.1016/j.pneurobio.2004.09.002 15556288

[jsr14462-bib-0025] Chang, C. , Cunningham, J. P. , & Glover, G. H. (2009). Influence of heart rate on the BOLD signal: The cardiac response function. NeuroImage, 44(3), 857–869. 10.1016/j.neuroimage.2008.09.029 18951982 PMC2677820

[jsr14462-bib-0026] Chowdhury, M. E. H. , Mullinger, K. J. , Glover, P. , & Bowtell, R. (2014). Reference layer artefact subtraction (RLAS): A novel method of minimizing EEG artefacts during simultaneous fMRI. NeuroImage, 84, 307–319. 10.1016/j.neuroimage.2013.08.039 23994127

[jsr14462-bib-0027] Colrain, I. M. (2005). The K‐complex: A 7‐decade history. Sleep, 28(2), 255–273. 10.1093/sleep/28.2.255 16171251

[jsr14462-bib-0028] Cooley, C. Z. , McDaniel, P. C. , Stockmann, J. P. , Srinivas, S. A. , Cauley, S. F. , Śliwiak, M. , Sappo, C. R. , Vaughn, C. F. , Guerin, B. , Rosen, M. S. , Lev, M. H. , & Wald, L. L. (2021). A portable scanner for magnetic resonance imaging of the brain. Nature Biomedical Engineering, 5(3), 229–239. 10.1038/s41551-020-00641-5 PMC8597947 33230306

[jsr14462-bib-0029] Corea, J. R. , Flynn, A. M. , Lechêne, B. , Scott, G. , Reed, G. D. , Shin, P. J. , Lustig, M. , & Arias, A. C. (2016). Screen‐printed flexible MRI receive coils. Nature Communications, 7(1) 10839. 10.1038/ncomms10839 PMC5553354 26961073

[jsr14462-bib-0030] Czisch, M. , Wehrle, R. , Kaufmann, C. , Wetter, T. C. , Holsboer, F. , Pollmächer, T. , & Auer, D. P. (2004). Functional MRI during sleep: BOLD signal decreases and their electrophysiological correlates. The European Journal of Neuroscience, 20(2), 566–574. 10.1111/j.1460-9568.2004.03518.x 15233766

[jsr14462-bib-0031] Czisch, M. , Wehrle, R. , Stiegler, A. , Peters, H. , Andrade, K. , Holsboer, F. , & Sämann, P. G. (2009). Acoustic oddball during NREM sleep: A combined EEG/fMRI study. PLoS One, 4(8), e6749. 10.1371/journal.pone.0006749 19707599 PMC2727699

[jsr14462-bib-0032] Czisch, M. , Wetter, T. C. , Kaufmann, C. , Pollmächer, T. , Holsboer, F. , & Auer, D. P. (2002). Altered processing of acoustic stimuli during sleep: Reduced auditory activation and visual deactivation detected by a combined fMRI/EEG study. NeuroImage, 16(1), 251–258. 11969332 10.1006/nimg.2002.1071

[jsr14462-bib-0033] Dang‐Vu, T. T. , Bonjean, M. , Schabus, M. , Boly, M. , Darsaud, A. , Desseilles, M. , Degueldre, C. , Balteau, E. , Phillips, C. , Luxen, A. , Sejnowski, T. J. , & Maquet, P. (2011). Interplay between spontaneous and induced brain activity during human non‐rapid eye movement sleep. Proceedings of the National Academy of Sciences of the United States of America, 108(37), 15438–15443. 10.1073/pnas.1112503108 21896732 PMC3174676

[jsr14462-bib-0034] Dang‐Vu, T. T. , Desseilles, M. , Laureys, S. , Degueldre, C. , Perrin, F. , Phillips, C. , Maquet, P. , & Peigneux, P. (2005). Cerebral correlates of delta waves during non‐REM sleep revisited. NeuroImage, 28(1), 14–21. 10.1016/j.neuroimage.2005.05.028 15979343

[jsr14462-bib-0035] Dang‐Vu, T. T. , Schabus, M. , Desseilles, M. , Albouy, G. , Boly, M. , Darsaud, A. , Gais, S. , Rauchs, G. , Sterpenich, V. , Vandewalle, G. , Carrier, J. , Moonen, G. , Balteau, E. , Degueldre, C. , Luxen, A. , Phillips, C. , & Maquet, P. (2008). Spontaneous neural activity during human slow wave sleep. Proceedings of the National Academy of Sciences of the United States of America, 105(39), 15160–15165. 10.1073/pnas.0801819105 18815373 PMC2567508

[jsr14462-bib-0036] De Gennaro, L. , & Ferrara, M. (2003). Sleep spindles: an overview. Sleep Medicine Reviews, 7(5), 423–440. 14573378 10.1053/smrv.2002.0252

[jsr14462-bib-0037] Deco, G. , Hagmann, P. , Hudetz, A. G. , & Tononi, G. (2014). Modeling resting‐state functional networks when the cortex falls asleep: Local and global changes. Cerebral Cortex, 24(12), 3180–3194. 10.1093/cercor/bht176 23845770 PMC6317504

[jsr14462-bib-0038] Deco, G. , McIntosh, A. R. , Shen, K. , Hutchison, R. M. , Menon, R. S. , Everling, S. , Hagmann, P. , & Jirsa, V. K. (2014). Identification of optimal structural connectivity using functional connectivity and neural modeling. Journal of Neuroscience, 34(23), 7910–7916. 24899713 10.1523/JNEUROSCI.4423-13.2014 PMC6608269

[jsr14462-bib-0039] Detre, J. A. , Wang, J. , Wang, Z. , & Rao, H. (2009). Arterial spin‐labeled perfusion MRI in basic and clinical neuroscience. Current Opinion in Neurology, 22(4), 348–355. 10.1097/WCO.0b013e32832d9505 19491678

[jsr14462-bib-0040] Dresler, M. , Koch, S. P. , Wehrle, R. , Spoormaker, V. I. , Holsboer, F. , Steiger, A. , Sämann, P. G. , Obrig, H. , & Czisch, M. (2011). Dreamed movement elicits activation in the sensorimotor cortex. Current Biology, 21(21), 1833–1837. 22036177 10.1016/j.cub.2011.09.029

[jsr14462-bib-0041] Dresler, M. , Spoormaker, V. I. , Beitinger, P. , Czisch, M. , Kimura, M. , Steiger, A. , & Holsboer, F. (2014). Neuroscience‐driven discovery and development of sleep therapeutics. Pharmacology & Therapeutics, 141(3), 300–334. 10.1016/j.pharmthera.2013.10.012 24189488

[jsr14462-bib-0042] Dresler, M. , Wehrle, R. , Spoormaker, V. I. , Koch, S. P. , Holsboer, F. , Steiger, A. , Obrig, H. , Sämann, P. G. , & Czisch, M. (2012). Neural correlates of dream lucidity obtained from contrasting lucid versus non‐lucid REM sleep: A combined EEG/fMRI case study. Sleep, 35(7), 1017–1020. 10.5665/sleep.1974 22754049 PMC3369221

[jsr14462-bib-0043] Duyn, J. H. , Ozbay, P. S. , Chang, C. , & Picchioni, D. (2020). Physiological changes in sleep that affect fMRI inference. Current Opinion in Behavioral Sciences, 33, 42–50. 10.1016/j.cobeha.2019.12.007 32613032 PMC7328858

[jsr14462-bib-0044] Eichenlaub, J. B. , Nicolas, A. , Daltrozzo, J. , Redouté, J. , Costes, N. , & Ruby, P. (2014). Resting brain activity varies with dream recall frequency between subjects. Neuropsychopharmacology, 39(7), 1594–1602. 10.1038/npp.2014.6 24549103 PMC4023156

[jsr14462-bib-0045] Eide, P. K. , Vinje, V. , Pripp, A. H. , Mardal, K. A. , & Ringstad, G. (2021). Sleep deprivation impairs molecular clearance from the human brain. Brain, 144(3), 863–874. 10.1093/brain/awaa443 33829232

[jsr14462-bib-0046] Farthouat, J. , & Peigneux, P. (2015). Memory reactivation in humans (imaging studies). In M. Tatsuno (Ed.), Analysis and modeling of coordinated multi‐neuronal activity (Vol. 12. Springer Series in Computational Neuroscience., pp. 225–243). Springer. 10.1007/978-1-4939-1969-7_11

[jsr14462-bib-0047] Fernandez, L. M. , & Lüthi, A. (2020). Sleep spindles: Mechanisms and functions. Physiological Reviews, 100(2), 805–868. 31804897 10.1152/physrev.00042.2018

[jsr14462-bib-0048] Filevich, E. , Dresler, M. , Brick, T. R. , & Kuhn, S. (2015). Metacognitive mechanisms underlying lucid dreaming. Journal of Neuroscience, 35(3), 1082–1088. 10.1523/JNEUROSCI.3342-14.2015 25609624 PMC6605529

[jsr14462-bib-0049] Finelli, L. A. , Borbély, A. A. , & Achermann, P. (2001). Functional topography of the human nonREM sleep electroencephalogram. European Journal of Neuroscience, 13(12), 2282–2290. 11454032 10.1046/j.0953-816x.2001.01597.x

[jsr14462-bib-0050] Frey, M. , Nau, M. , & Doeller, C. F. (2021). Magnetic resonance‐based eye tracking using deep neural networks. Nature Neuroscience, 24(12), 1772–1779. 10.1038/s41593-021-00947-w 34750593 PMC10097595

[jsr14462-bib-0051] Fultz, N. E. , Bonmassar, G. , Setsompop, K. , Stickgold, R. A. , Rosen, B. R. , Polimeni, J. R. , & Lewis, L. D. (2019). Coupled electrophysiological, hemodynamic, and cerebrospinal fluid oscillations in human sleep. Science, 366(6465), 628–631. 10.1126/science.aax5440 31672896 PMC7309589

[jsr14462-bib-0052] Gott, J. A. , Liley, D. T. , & Hobson, J. A. (2017). Towards a functional understanding of PGO waves. Frontiers in Human Neuroscience, 11, 89. 28316568 10.3389/fnhum.2017.00089 PMC5334507

[jsr14462-bib-0053] Hale, J. R. , White, T. P. , Mayhew, S. D. , Wilson, R. S. , Rollings, D. T. , Khalsa, S. , Arvanitis, T. N. , & Bagshaw, A. P. (2016). Altered thalamocortical and intra‐thalamic functional connectivity during light sleep compared with wake. NeuroImage, 125, 657–667. 10.1016/j.neuroimage.2015.10.041 26499809

[jsr14462-bib-0054] Happe, S. , Anderer, P. , Gruber, G. , Klösch, G. , Saletu, B. , & Zeitlhofer, J. (2002). Scalp topography of the spontaneous K‐complex and of delta‐waves in human sleep. Brain Topography, 15(1), 43–49. 12371676 10.1023/a:1019992523246

[jsr14462-bib-0055] Hobson, J. (1964). L'activité électrique phasique du cortex et du thalamus au cours du sommeil désynchronisé chez le chat. Comptes Rendus des Seances de la Societe de Biologie et de Ses Filiales, 158, 2131–2135. 14282131

[jsr14462-bib-0056] Hofle, N. , Paus, T. , Reutens, D. , Fiset, P. , Gotman, J. , Evans, A. C. , & Jones, B. E. (1997). Regional cerebral blood flow changes as a function of Delta and spindle activity during slow wave sleep in humans. The Journal of Neuroscience, 17(12), 4800–4808. 10.1523/JNEUROSCI.17-12-04800.1997 9169538 PMC6573353

[jsr14462-bib-0057] Hong, C. , Fallon, J. , & Friston, K. (2021). fMRI evidence for default mode network deactivation associated with rapid eye movements in sleep. Brain Sciences, 11(11) 1528. 10.3390/brainsci11111528 PMC8615877 34827529

[jsr14462-bib-0058] Hong, C. C. , Potkin, S. G. , Antrobus, J. S. , Dow, B. M. , Callaghan, G. M. , & Gillin, J. C. (1997). REM sleep eye movement counts correlate with visual imagery in dreaming: A pilot study. Psychophysiology, 34(3), 377–381. 9175452 10.1111/j.1469-8986.1997.tb02408.x

[jsr14462-bib-0059] Hong, C. C. H. , Harris, J. C. , Pearlson, G. D. , Kim, J. S. , Calhoun, V. D. , Fallon, J. H. , Golay, X. , Gillen, J. S. , Simmonds, D. J. , van Zijl, P. C. M. , Zee, D. S. , & Pekar, J. J. (2009). FMRI evidence for multisensory recruitment associated with rapid eye movements during sleep. Human Brain Mapping, 30(5), 1705–1722. 10.1002/hbm.20635 18972392 PMC2753360

[jsr14462-bib-0060] Horovitz, S. G. , Braun, A. R. , Carr, W. S. , Picchioni, D. , Balkin, T. J. , Fukunaga, M. , & Duyn, J. H. (2009). Decoupling of the brain's default mode network during deep sleep. National Academy of Sciences of the United States of America, 106(27), 11376–11381. 10.1073/pnas.0901435106 PMC2708777 19549821

[jsr14462-bib-0061] Horovitz, S. G. , Fukunaga, M. , De Zwart, J. A. , van Gelderen, P. , Fulton, S. C. , Balkin, T. J. , & Duyn, J. H. (2008). Low frequency BOLD fluctuations during resting wakefulness and light sleep: A simultaneous EEG‐fMRI study. Human Brain Mapping, 29(6), 671–682. 10.1002/hbm.20428 17598166 PMC6871022

[jsr14462-bib-0062] Huettel, S. , Song, A. , & McCarthy, G. (2004). Functional magnetic resonance imaging. Sinauer associates. Inc. Published online (pp. 162–170). Sinauer Associates

[jsr14462-bib-0063] Ido, T. , Wan, C. , Casella, V. , Fowler, J. S. , Wolf, A. P. , Reivich, M. , & Kuhl, D. E. (1978). Labeled 2‐deoxy‐D‐glucose analogs. 18F‐labeled 2‐deoxy‐2‐fluoro‐D‐glucose, 2‐deoxy‐2‐fluoro‐D‐mannose and 14C‐2‐deoxy‐2‐fluoro‐D‐glucose. Journal of Labelled Compounds and Radiopharmaceuticals, 14(2), 175–183.

[jsr14462-bib-0064] Ioannides, A. A. , Kostopoulos, G. K. , Liu, L. , & Fenwick, P. B. C. (2009). MEG identifies dorsal medial brain activations during sleep. NeuroImage, 44(2), 455–468. 10.1016/j.neuroimage.2008.09.030 18950718

[jsr14462-bib-0065] Jahnke, K. , von Wegner, F. , Morzelewski, A. , Borisov, S. , Maischein, M. , Steinmetz, H. , & Laufs, H. (2012). To wake or not to wake? The two‐sided nature of the human K‐complex. NeuroImage, 59(2), 1631–1638. 10.1016/j.neuroimage.2011.09.013 21945697

[jsr14462-bib-0066] Jiang, J. , Zou, G. , Liu, J. , Zhou, S. , Xu, J. , Sun, H. , Zou, Q. , & Gao, J. H. (2021:hbm.25461). Functional connectivity of the human hypothalamus during wakefulness and nonrapid eye movement sleep. Human Brain Mapping, 42, 3667–3679. 10.1002/hbm.25461 33960583 PMC8249893

[jsr14462-bib-0067] Jouvet, M. (1959). Corrélations électromyographiques du sommeil chez le chat décortiqué et mésencéphalique chronique. Comptes Rendus des Seances de la Societe de Biologie et de Ses Filiales, 153, 422–425. 13663472

[jsr14462-bib-0068] Kajimura, N. , Uchiyama, M. , Takayama, Y. , Uchida, S. , Uema, T. , Kato, M. , Sekimoto, M. , Watanabe, T. , Nakajima, T. , Horikoshi, S. , Ogawa, K. , Nishikawa, M. , Hiroki, M. , Kudo, Y. , Matsuda, H. , Okawa, M. , & Takahashi, K. (1999). Activity of midbrain reticular formation and neocortex during the progression of human non‐rapid eye movement sleep. The Journal of Neuroscience, 19(22), 10065–10073. 10.1523/JNEUROSCI.19-22-10065.1999 10559414 PMC6782956

[jsr14462-bib-0069] Khalsa, S. , Hale, J. R. , Goldstone, A. , Wilson, R. S. , Mayhew, S. D. , Bagary, M. , & Bagshaw, A. P. (2017). Habitual sleep durations and subjective sleep quality predict white matter differences in the human brain. Neurobiology of Sleep and Circadian Rhythms, 3, 17–25. 31236500 10.1016/j.nbscr.2017.03.001 PMC6575574

[jsr14462-bib-0070] Koike, T. , Kan, S. , Misaki, M. , & Miyauchi, S. (2011). Connectivity pattern changes in default‐mode network with deep non‐REM and REM sleep. Neuroscience Research, 69(4), 322–330. 10.1016/j.neures.2010.12.018 21238510

[jsr14462-bib-0071] Kwong, K. K. , Belliveau, J. W. , Chesler, D. A. , Goldberg, I. E. , Weisskoff, R. M. , Poncelet, B. P. , Kennedy, D. N. , Hoppel, B. E. , Cohen, M. S. , & Turner, R. (1992). Dynamic magnetic resonance imaging of human brain activity during primary sensory stimulation. National Academy of Sciences of the United States of America, 89(12), 5675–5679. 10.1073/pnas.89.12.5675 PMC49355 1608978

[jsr14462-bib-0072] Larson‐Prior, L. J. , Power, J. D. , Vincent, J. L. , Nolan, T. S. , Coalson, R. S. , Zempel, J. , Snyder, A. Z. , Schlaggar, B. L. , Raichle, M. E. , & Petersen, S. E. (2011). Modulation of the brain's functional network architecture in the transition from wake to sleep. Progress in Brain Research, 193, 277–294. 10.1016/B978-0-444-53839-0.00018-1 Elsevier. 21854969 PMC3811144

[jsr14462-bib-0073] Larson‐Prior, L. J. , Zempel, J. M. , Nolan, T. S. , Prior, F. W. , Snyder, A. , & Raichle, M. E. (2009). Cortical network functional connectivity in the descent to sleep. Proceedings of the National Academy of Sciences of the United States of America, 106(11), 4489–4494. 10.1073/pnas.0900924106 19255447 PMC2657465

[jsr14462-bib-0074] Laufs, H. , Walker, M. C. , & Lund, T. E. (2007). ’Brain activation and hypothalamic functional connectivity during human non‐rapid eye movement sleep: An EEG/fMRI study’‐‐its limitations and an alternative approach. Brain, 130(7) e75‐e75, e75. 10.1093/brain/awm084 17584775

[jsr14462-bib-0075] Levitt, J. , Yang, Z. , & Lewis, L. D. (2022:2022.11.21.515651). EEG‐LLAMAS: An open source, low latency, EEG‐fMRI neurofeedback platform. doi:10.1101/2022.11.21.515651

[jsr14462-bib-0076] Lewis, L. D. (2021). The interconnected causes and consequences of sleep in the brain. Science, 374(6567), 564–568. 10.1126/science.abi8375 34709917 PMC8815779

[jsr14462-bib-0077] Liebig, P. , Heidemann, R. M. , Hensel, B. , & Porter, D. A. (2019). Accelerated silent echo‐planar imaging. Magnetic Resonance Imaging, 55, 81–85. 10.1016/j.mri.2018.09.016 30236603

[jsr14462-bib-0078] Loomis, A. L. , Harvey, E. N. , & Hobart, G. (1935). Potential rhythms of the cerebral cortex during sleep. Science, 81(2111), 597–598. 17739875 10.1126/science.81.2111.597

[jsr14462-bib-0079] Lövblad, K. O. , Thomas, R. , Jakob, P. M. , Scammell, T. , Bassetti, C. , Griswold, M. , Ives, J. , Matheson, J. , Edelman, R. R. , & Warach, S. (1999). Silent functional magnetic resonance imaging demonstrates focal activation in rapid eye movement sleep. Neurology, 53(9), 2193. 10.1212/WNL.53.9.2193 10599807

[jsr14462-bib-0080] Madsen, P. L. , Schmidt, J. F. , Wildschiødtz, G. , Friberg, L. , Holm, S. , Vorstrup, S. , & Lassen, N. A. (1991). Cerebral O_2_ metabolism and cerebral blood flow in humans during deep and rapid‐eye‐movement sleep. Journal of Applied Physiology (1985), 70(6), 2597–2601. 10.1152/jappl.1991.70.6.2597 1885454

[jsr14462-bib-0081] Mander, B. A. , Zhu, A. H. , Lindquist, J. R. , Villeneuve, S. , Rao, V. , Lu, B. , Saletin, J. M. , Ancoli‐Israel, S. , Jagust, W. J. , & Walker, M. P. (2017). White matter structure in older adults moderates the benefit of sleep spindles on motor memory consolidation. The Journal of Neuroscience, 37(48), 11675–11687. 10.1523/JNEUROSCI.3033-16.2017 29084867 PMC5707766

[jsr14462-bib-0082] Maquet, P. , Dive, D. , Salmon, E. , Sadzot, B. , Franco, G. , Poirrier, R. , von Frenckell, R. , & Franck, G. (1990). Cerebral glucose utilization during sleep‐wake cycle in man determined by positron emission tomography and [18f]2‐Fluoro‐2‐deoxy‐D‐glucose method. Brain Research, 513(1), 136–143. 10.1016/0006-8993(90)91099-3 2350676

[jsr14462-bib-0083] Maquet, P. , Dive, D. , Salmon, E. , Sadzot, B. , Franco, G. , Poirrier, R. , & Franck, G. (1992). Cerebral glucose utilization during stage 2 sleep in man. Brain Research, 571(1), 149–153. 10.1016/0006-8993(92)90522-B 1611488

[jsr14462-bib-0084] Maquet, P. , Péters, J. M. , Aerts, J. , Delfiore, G. , Degueldre, C. , Luxen, A. , & Franck, G. (1996). Functional neuroanatomy of human rapid‐eye‐movement sleep and dreaming. Nature, 383(6596), 163–166. 8774879 10.1038/383163a0

[jsr14462-bib-0085] McCormick, D. A. , & Bal, T. (1994). Sensory gating mechanisms of the thalamus. Current Opinion in Neurobiology, 4(4), 550–556. 10.1016/0959-4388(94)90056-6 7812144

[jsr14462-bib-0086] McGaugh, J. L. (2013). Making lasting memories: Remembering the significant. Proceedings of the National Academy of Sciences, 110(supplement_2), 10402–10407. 10.1073/pnas.1301209110 PMC3690616 23754441

[jsr14462-bib-0087] Mikiten, T. (1961). EEG desynchronization during behavioral sleep associated with spike discharges from the thalamus of the cat. In, 20, 327.

[jsr14462-bib-0088] Mitra, A. , Snyder, A. Z. , Tagliazucchi, E. , Laufs, H. , & Raichle, M. E. (2015). Propagated infra‐slow intrinsic brain activity reorganizes across wake and slow wave sleep. eLife, 4, e10781. 10.7554/eLife.10781 26551562 PMC4737658

[jsr14462-bib-0089] Miyauchi, S. , Misaki, M. , Kan, S. , Fukunaga, T. , & Koike, T. (2009). Human brain activity time‐locked to rapid eye movements during REM sleep. Experimental Brain Research, 192(4), 657–667. 10.1007/s00221-008-1579-2 18830586

[jsr14462-bib-0090] Moehlman, T. M. , de Zwart, J. A. , Chappel‐Farley, M. G. , Liu, X. , McClain, I. B. , Chang, C. , Mandelkow, H. , Özbay, P. S. , Johnson, N. L. , Bieber, R. E. , Fernandez, K. A. , King, K. A. , Zalewski, C. K. , Brewer, C. C. , van Gelderen, P. , Duyn, J. H. , & Picchioni, D. (2019). All‐night functional magnetic resonance imaging sleep studies. Journal of Neuroscience Methods, 316, 83–98. 10.1016/j.jneumeth.2018.09.019 30243817 PMC6524535

[jsr14462-bib-0091] Mouret, J. , Jeannerod, M. , & Jouvet, M. (1963). Lactivite electrique du systeme visuel au cours de la phase paradoxale du sommeil chez le chat. Journal de Physiologie, 55(2), 305. 14219833

[jsr14462-bib-0092] Mulert, C. , & Lemieux, L. (2009). EEG ‐ fMRI: Physiological basis, technique, and applications. Springer Science & Business Media.

[jsr14462-bib-0093] Naji, M. , Krishnan, G. P. , McDevitt, E. A. , Bazhenov, M. , & Mednick, S. C. (2019). Coupling of autonomic and central events during sleep benefits declarative memory consolidation. Neurobiology of Learning and Memory, 157, 139–150. 10.1016/j.nlm.2018.12.008 30562589 PMC6425961

[jsr14462-bib-0094] Özbay, P. S. , Chang, C. , Picchioni, D. , Mandelkow, H. , Chappel‐Farley, M. G. , van Gelderen, P. , de Zwart, J. A. , & Duyn, J. (2019). Sympathetic activity contributes to the fMRI signal. Communications Biology, 2(1), 1–9. 10.1038/s42003-019-0659-0 31754651 PMC6861267

[jsr14462-bib-0095] Peigneux, P. , Laureys, S. , Fuchs, S. , Delbeuck, X. , Degueldre, C. , Aerts, J. , Delfiore, G. , Luxen, A. , & Maquet, P. (2001). Generation of rapid eye movements during paradoxical sleep in humans. NeuroImage, 14(3), 701–708. 10.1006/nimg.2001.0874 11506542

[jsr14462-bib-0096] Phelps, M. E. , Hoffman, E. J. , Mullani, N. A. , & Ter‐Pogossian, M. M. (1975). Application of annihilation coincidence detection to transaxial reconstruction tomography. Journal of Nuclear Medicine, 16(3), 210–224. 1113170

[jsr14462-bib-0097] Piantoni, G. , Poil, S. S. , Linkenkaer‐Hansen, K. , Verweij, I. M. , Ramautar, J. R. , van Someren, E. J. W. , & van der Werf, Y. D. (2013). Individual differences in white matter diffusion affect sleep oscillations. Journal of Neuroscience, 33(1), 227–233. 23283336 10.1523/JNEUROSCI.2030-12.2013 PMC6618630

[jsr14462-bib-0098] Picchioni, D. , Fukunaga, M. , Carr, W. S. , Braun, A. R. , Balkin, T. J. , Duyn, J. H. , & Horovitz, S. G. (2008). fMRI differences between early and late stage‐1 sleep. Neuroscience Letters, 441, 5–85. 10.1016/j.neulet.2008.06.010 PMC2670064 18584959

[jsr14462-bib-0099] Picchioni, D. , Özbay, P. S. , Mandelkow, H. , de Zwart, J. A. , Wang, Y. , van Gelderen, P. , & Duyn, J. H. (2022). Autonomic arousals contribute to brain fluid pulsations during sleep. NeuroImage, 249, 118888. 10.1016/j.neuroimage.2022.118888 35017126 PMC11395500

[jsr14462-bib-0100] Picchioni, D. , Pixa, M. L. , Fukunaga, M. , Carr, W. S. , Horovitz, S. G. , Braun, A. R. , & Duyn, J. H. (2014). Decreased connectivity between the thalamus and the neocortex during human nonrapid eye movement sleep. Sleep, 37(2), 387–397. 10.5665/sleep.3422 24497667 PMC3900615

[jsr14462-bib-0101] Portas, C. M. , Krakow, K. , Allen, P. , Josephs, O. , Armony, J. L. , & Frith, C. D. (2000). Auditory processing across the sleep‐wake cycle: Simultaneous EEG and fMRI monitoring in humans. Neuron, 28(3), 991–999. 11163282 10.1016/s0896-6273(00)00169-0

[jsr14462-bib-0102] Redcay, E. , Kennedy, D. P. , & Courchesne, E. (2007). fMRI during natural sleep as a method to study brain function during early childhood. NeuroImage, 38(4), 696–707. 17904385 10.1016/j.neuroimage.2007.08.005

[jsr14462-bib-0103] Saletin, J. M. , van der Helm, E. , & Walker, M. P. (2013). Structural brain correlates of human sleep oscillations. NeuroImage, 83, 658–668. 23770411 10.1016/j.neuroimage.2013.06.021 PMC4263481

[jsr14462-bib-0104] Sämann, P. G. , Wehrle, R. , Hoehn, D. , Spoormaker, V. I. , Peters, H. , Tully, C. , Holsboer, F. , & Czisch, M. (2011). Development of the Brain's default mode network from wakefulness to slow wave sleep. Cerebral Cortex, 21(9), 2082–2093. 10.1093/cercor/bhq295 21330468

[jsr14462-bib-0105] Schabus, M. , Dang‐Vu, T. T. , Albouy, G. , Balteau, E. , Boly, M. , Carrier, J. , Darsaud, A. , Degueldre, C. , Desseilles, M. , Gais, S. , Phillips, C. , Rauchs, G. , Schnakers, C. , Sterpenich, V. , Vandewalle, G. , Luxen, A. , & Maquet, P. (2007). Hemodynamic cerebral correlates of sleep spindles during human non‐rapid eye movement. Sleep, 104, 13164–13169. 10.1073/pnas.0703084104 PMC1941810 17670944

[jsr14462-bib-0106] Schabus, M. , Dang‐Vu, T. T. , Heib, D. , Boly, M. , Desseilles, M. , Vandewalle, G. , Schmidt, C. , Albouy, G. , Darsaud, A. , Gais, S. , Degueldre, C. , Balteau, E. , Phillips, C. , Luxen, A. , & Maquet, P. (2012). The fate of incoming stimuli during NREM sleep is determined by spindles and the phase of the slow oscillation. Frontiers in Neurology, 3, 40. Accessed October 25, 2022. 10.3389/fneur.2012.00040 22493589 PMC3319907

[jsr14462-bib-0107] Schmitter, S. , Diesch, E. , Amann, M. , Kroll, A. , Moayer, M. , & Schad, L. R. (2008). Silent echo‐planar imaging for auditory FMRI. Magnetic Resonance Materials in Physics, Biology and Medicine, 21(5), 317–325. 10.1007/s10334-008-0132-4 18716815

[jsr14462-bib-0108] Setzer, B. , Fultz, N. E. , Gomez, D. E. P. , Williams, S. D. , Bonmassar, G. , Polimeni, J. R. , & Lewis, L. D. (2022). A temporal sequence of thalamic activity unfolds at transitions in behavioral arousal state. Nature Communications, 13(1), 5442. 10.1038/s41467-022-33010-8 PMC9481532 36114170

[jsr14462-bib-0109] Shmueli, K. , van Gelderen, P. , de Zwart, J. A. , Horovitz, S. G. , Fukunaga, M. , Jansma, J. M. , & Duyn, J. H. (2007). Low‐frequency fluctuations in the cardiac rate as a source of variance in the resting‐state fMRI BOLD signal. NeuroImage, 38(2), 306–320. 10.1016/j.neuroimage.2007.07.037 17869543 PMC2128785

[jsr14462-bib-0110] Siclari, F. , Bernardi, G. , Riedner, B. A. , LaRocque, J. J. , Benca, R. M. , & Tononi, G. (2014). Two distinct synchronization processes in the transition to sleep: A high‐density electroencephalographic study. Sleep, 37(10), 1621–1637. 25197810 10.5665/sleep.4070 PMC4173919

[jsr14462-bib-0111] Simor, P. , van der Wijk, G. , Nobili, L. , & Peigneux, P. (2020). The microstructure of REM sleep: Why phasic and tonic? Sleep Medicine Reviews, 52, 101305. 10.1016/j.smrv.2020.101305 32259697

[jsr14462-bib-0112] Solms, M. (1997). What is consciousness? Journal of the American Psychoanalytic Association, 45(3), 681–703. 9353705 10.1177/00030651970450031201

[jsr14462-bib-0113] Song, C. , Boly, M. , Tagliazucchi, E. , Laufs, H. , & Tononi, G. (2022). fMRI spectral signatures of sleep. National Academy of Sciences of the United States of America, 119(30), e2016732119. 10.1073/pnas.2016732119 PMC9335231 35862450

[jsr14462-bib-0114] Song, C. , & Tagliazucchi, E. (2020). Linking the nature and functions of sleep: Insights from multimodal imaging of the sleeping brain. Current Opinion in Physiology, 15, 29–36. 10.1016/j.cophys.2019.11.012 32715184 PMC7374576

[jsr14462-bib-0115] Soon, C. S. , Vinogradova, K. , Ong, J. L. , Calhoun, V. D. , Liu, T. , Zhou, J. H. , Ng, K. K. , & Chee, M. W. L. (2021). Respiratory, cardiac, EEG, BOLD signals and functional connectivity over multiple microsleep episodes. NeuroImage, 237, 118129. 10.1016/j.neuroimage.2021.118129 33951513

[jsr14462-bib-0116] Spoormaker, V. I. , Gleiser, P. M. , & Czisch, M. (2012). Frontoparietal connectivity and hierarchical structure of the brain's functional network during sleep. Frontiers in Neurology, 3, MAY. 10.3389/fneur.2012.00080 PMC3354331 22629253

[jsr14462-bib-0117] Spoormaker, V. I. , Schröter, M. S. , Gleiser, P. M. , Andrade, K. C. , Dresler, M. , Wehrle, R. , Sämann, P. G. , & Czisch, M. (2010). Development of a large‐scale functional brain network during human non‐rapid eye movement sleep. Journal of Neuroscience, 30(34), 11379–11387. 10.1523/JNEUROSCI.2015-10.2010 20739559 PMC6633325

[jsr14462-bib-0118] Stee, W. , & Peigneux, P. (2021). Post‐learning micro‐and macro‐structural neuroplasticity changes with time and sleep. Biochemical Pharmacology, 191, 114369. 33338474 10.1016/j.bcp.2020.114369

[jsr14462-bib-0119] Steriade, M. , & McCarley, R. W. (2005). Synchronized brain oscillations leading to neuronal plasticity during waking and sleep states. Brain Control of Wakefulness and Sleep, 255–344. Springer.

[jsr14462-bib-0120] Steriade, M. M. , & McCarley, R. W. (2013). Brainstem control of wakefulness and sleep. Springer Science & Business Media.

[jsr14462-bib-0121] Stern, J. M. , Caporro, M. , Haneef, Z. , Yeh, H. J. , Buttinelli, C. , Lenartowicz, A. , Mumford, J. A. , Parvizi, J. , & Poldrack, R. A. (2011). Functional imaging of sleep vertex sharp transients. Clinical Neurophysiology, 122(7), 1382–1386. 21310653 10.1016/j.clinph.2010.12.049 PMC3105179

[jsr14462-bib-0122] Stevner, A. B. A. , Vidaurre, D. , Cabral, J. , Rapuano, K. , Nielsen, S. F. V. , Tagliazucchi, E. , Laufs, H. , Vuust, P. , Deco, G. , Woolrich, M. W. , Van Someren, E. , & Kringelbach, M. L. (2019). Discovery of key whole‐brain transitions and dynamics during human wakefulness and non‐REM sleep. Nature Communications, 10(1), 1035. 10.1038/s41467-019-08934-3 PMC6399232 30833560

[jsr14462-bib-0123] Sun, H. , Ganglberger, W. , Panneerselvam, E. , Leone, M. J. , Quadri, S. A. , Goparaju, B. , Tesh, R. A. , Akeju, O. , Thomas, R. J. , & Westover, M. B. (2020). Sleep staging from electrocardiography and respiration with deep learning. Sleep, 43(7), zsz306. 10.1093/sleep/zsz306 31863111 PMC7355395

[jsr14462-bib-0124] Tagliazucchi, E. , Crossley, N. , Bullmore, E. T. , & Laufs, H. (2016). Deep sleep divides the cortex into opposite modes of anatomical–functional coupling. Brain Structure and Function, 221(8), 4221–4234. 10.1007/s00429-015-1162-0 26650048

[jsr14462-bib-0125] Tagliazucchi, E. , Von Wegner, F. , Morzelewski, A. , Brodbeck, V. , Jahnke, K. , & Laufs, H. (2013). Breakdown of long‐range temporal dependence in default mode and attention networks during deep sleep. Proceedings of the National Academy of Sciences of the United States of America, 110(38), 15419–15424. 10.1073/pnas.1312848110 24003146 PMC3780893

[jsr14462-bib-0126] Takeuchi, H. , Taki, Y. , Nouchi, R. , Yokoyama, R. , Kotozaki, Y. , Nakagawa, S. , Sekiguchi, A. , Iizuka, K. , Yamamoto, Y. , Hanawa, S. , Araki, T. , Miyauchi, C. M. , Shinada, T. , Sakaki, K. , Nozawa, T. , Ikeda, S. , Yokota, S. , Daniele, M. , Sassa, Y. , & Kawashima, R. (2018). Shorter sleep duration and better sleep quality are associated with greater tissue density in the brain. Scientific Reports, 8(1), 1–8. 29643448 10.1038/s41598-018-24226-0 PMC5895621

[jsr14462-bib-0127] Ter‐Pogossian, M. M. , Phelps, M. E. , Hoffman, E. J. , & Mullani, N. A. (1975). A positron‐emission transaxial tomograph for nuclear imaging (PETT). Radiology, 114(1), 89–98. 1208874 10.1148/114.1.89

[jsr14462-bib-0128] Titone, S. , Samogin, J. , Peigneux, P. , Swinnen, S. P. , Mantini, D. , & Albouy, G. (2024). Frequency‐dependent connectivity in large‐scale resting‐state brain networks during sleep. European Journal of Neuroscience, 59(4), 686–702. 10.1111/ejn.16080 37381891

[jsr14462-bib-0129] Tüshaus, L. , Omlin, X. , Tuura, R. O. , Federspiel, A. , Luechinger, R. , Staempfli, P. , Koenig, T. , & Achermann, P. (2017). In human non‐REM sleep, more slow‐wave activity leads to less blood flow in the prefrontal cortex. Scientific Reports, 7(1), 1–13. 29101338 10.1038/s41598-017-12890-7 PMC5670199

[jsr14462-bib-0130] Ujma, P. P. (2021). Sleep spindles and general cognitive ability–A meta‐analysis. Sleep Spindles & Cortical up States, 2(1), 1–17.

[jsr14462-bib-0131] Vallat, R. , Eichenlaub, J. B. , Nicolas, A. , & Ruby, P. (2018). Dream recall frequency is associated with medial prefrontal cortex White‐matter density. Frontiers in Psychology, 9, 416243. 10.3389/fpsyg.2018.01856 PMC6171441 30319519

[jsr14462-bib-0132] van der Meer, J. N. , Pampel, A. , Van Someren, E. J. W. , Ramautar, J. R. , van der Werf, Y. D. , Gomez‐Herrero, G. , Lepsien, J. , Hellrung, L. , Hinrichs, H. , Möller, H. E. , & Walter, M. (2016). Carbon‐wire loop based artifact correction outperforms post‐processing EEG/fMRI corrections—A validation of a real‐time simultaneous EEG/fMRI correction method. NeuroImage, 125, 880–894. 10.1016/j.neuroimage.2015.10.064 26505301

[jsr14462-bib-0133] Vanhaudenhuyse, A. , Noirhomme, Q. , Tshibanda, L. J. F. , Bruno, M.‐A. , Boveroux, P. , Schnakers, C. , Soddu, A. , Perlbarg, V. , Ledoux, D. , Brichant, J.‐F. , Moonen, G. , Maquet, P. , Greicius, M. D. , Laureys, S. , & Boly, M. (2010). Default network connectivity reflects the level of consciousness in non‐communicative brain‐damaged patients. Brain, 133(1), 161–171. 10.1093/brain/awp313 20034928 PMC2801329

[jsr14462-bib-0134] Voss, U. , Holzmann, R. , Tuin, I. , & Hobson, A. J. (2009). Lucid dreaming: A state of consciousness with features of both waking and non‐lucid dreaming. Sleep, 32(9), 1191–1200. 10.1093/sleep/32.9.1191 19750924 PMC2737577

[jsr14462-bib-0135] Wang, Y. J. , Duan, W. , & Lei, X. (2020). Impaired coupling of the Brain's default network during sleep deprivation: A resting‐state EEG study. Nature and Science of Sleep, 12, 937–947. 10.2147/NSS.S277655 PMC7667510 33204197

[jsr14462-bib-0136] Watanabe, T. , Kan, S. , Koike, T. , Misaki, M. , Konishi, S. , Miyauchi, S. , Miyahsita, Y. , & Masuda, N. (2014). Network‐dependent modulation of brain activity during sleep. NeuroImage, 98, 1–10. 10.1016/j.neuroimage.2014.04.079 24814208

[jsr14462-bib-0137] Wehrle, R. , Czisch, M. , Kaufmann, C. , Wetter, T. C. , Holsboer, F. , Auer, D. P. , & Pollmächer, T. (2005). Rapid eye movement‐related brain activation in human sleep: A functional magnetic resonance imaging study. Neuroreport, 16, no. 8, 853–857. 15891584 10.1097/00001756-200505310-00015

[jsr14462-bib-0138] Werth, E. , Achermann, P. , & Borbély, A. (1997). Fronto‐occipital EEG power gradients in human sleep. Journal of Sleep Research, 6(2), 102–112. 9377529 10.1046/j.1365-2869.1997.d01-36.x

[jsr14462-bib-0139] Wilf, M. , Ramot, M. , Furman‐Haran, E. , Arzi, A. , Levkovitz, Y. , & Malach, R. (2016). Diminished auditory responses during NREM sleep correlate with the hierarchy of language processing. PLoS One, 11(6), e0157143. 27310812 10.1371/journal.pone.0157143 PMC4911044

[jsr14462-bib-0140] Wilke, M. , Holland, S. K. , & Ball, W. S. (2003). Language processing during natural sleep in a 6‐year‐old boy, as assessed with functional MR imaging. American Journal of Neuroradiology, 24(1), 42–44. 12533325 PMC1351213

[jsr14462-bib-0141] Wu, C. W. , Liu, P. Y. , Tsai, P. J. , Wu, Y. C. , Hung, C. S. , Tsai, Y. C. , Cho, K. H. , Biswal, B. B. , Chen, C. J. , & Lin, C. P. (2012). Variations in connectivity in the sensorimotor and default‐mode networks during the first nocturnal sleep cycle. Brain Connectivity, 2(4), 177–190. 10.1089/brain.2012.0075 22817652

[jsr14462-bib-0142] Xie, L. , Kang, H. , Xu, Q. , Chen, M. J. , Liao, Y. , Thiyagarajan, M. , O'Donnell, J. , Christensen, D. J. , Nicholson, C. , Iliff, J. J. , Takano, T. , Deane, R. , & Nedergaard, M. (2013). Sleep drives metabolite clearance from the adult brain. Science, 342(6156), 373–377. 10.1126/science.1241224 24136970 PMC3880190

[jsr14462-bib-0143] Xu, D. , Chen, X. , Tian, Y. , Wan, X. , & Lei, X. (2021). Lying posture affects sleep structures and cortical activities: A simultaneous EEG‐fMRI imaging of the sleeping and waking brain. Brain Imaging and Behavior, 15(4), 2178–2186. 10.1007/s11682-020-00413-4 33215251

[jsr14462-bib-0144] Yang, F. N. , Picchioni, D. , de Zwart, J. A. , Wang, Y. , van Gelderen, P. , & Duyn, J. H. (2024). Reproducible, data‐driven characterization of sleep based on brain dynamics and transitions from whole‐night fMRI. Behrens TE, ed. eLife, 13:RP98739. doi:10.7554/eLife.98739 39331523 PMC11434609

[jsr14462-bib-0145] Zhang, H. , Schneider, T. , Wheeler‐Kingshott, C. A. , & Alexander, D. C. (2012). NODDI: Practical in vivo neurite orientation dispersion and density imaging of the human brain. NeuroImage, 61(4), 1000–1016. 22484410 10.1016/j.neuroimage.2012.03.072

